# Oral Recombinant Human Collagen III Protects Gastric Mucosa via G‐Quadruplex‐Mediated Activation of the Pla2g4a‐Prostaglandin Axis

**DOI:** 10.1002/advs.77964

**Published:** 2026-09-27

**Authors:** Xinyue Lan, Qiu Dai, Yanhui Wang, Jiaxuan Gu, Chengyun Wang, Weicheng Xu, Xinru Gao, Jiageng Cheng, Wentao Geng, Xiaowen Wu, Haihang Li, Xiaoyun He, Shengwei Fu, Longjiao Zhu, Wentao Xu

**Affiliations:** ^1^ Food Laboratory of Zhongyuan Key Laboratory of Precision Nutrition and Food Quality Department of Nutrition and Health China Agricultural University Beijing People's Republic of China; ^2^ Key Laboratory of Precision Nutrition and Food Quality Beijing Laboratory for Food Quality and Safety College of Food Science and Nutritional Engineering China Agricultural University Beijing People's Republic of China; ^3^ Jiangsu Trautec Medical Technology Co., Ltd Jiangsu People's Republic of China

**Keywords:** Ethanol‐induced acute gastric mucosal injury, Gastric mucosal protection, G‐quadruplex, Metabolic reprogramming, *Pla2g4a*‐prostaglandin axis, Recombinant human collagen III

## Abstract

Ethanol‐induced acute gastric mucosal injury (AGMI) remains a prevalent clinical problem, while current therapies are constrained by adverse effects and the immunogenicity of animal‐derived collagens. Here, we evaluate orally administered recombinant human type III collagen (RC) as a gastroprotective biomaterial. Following oral administration, RC reduced gastric mucosal lesion area by 78% within the dynamic acidic gastric environment, provided approximately 1.5‐fold greater protection than commercial animal‐derived collagens, and exhibited excellent biosafety. Integrated metabolomic and transcriptomic analyses revealed coordinated regulation of glutathione‐centered redox homeostasis, cytoskeletal and tight‐junction barrier repair, and metabolic‐inflammatory networks. Targeted LC‐MS/MS confirmed the transient occurrence of the RC‐derived pentapeptide GPAEF in gastric mucosal lavage 30 min after administration. GPAEF selectively recognized and stabilized the Pla2g4a promoter G‐quadruplex (G4), exhibiting approximately 13‐fold higher affinity than ThT and enhancing G4 folding under physiologically relevant ionic conditions. Molecular dynamics simulations indicated that GPAEF shifted the Pla2g4a G4 from a preferentially parallel topology toward hybrid/mixed conformations and stabilized the peptide‐G4 complex, providing a dynamic basis for Pla2g4a transcription and downstream prostaglandin biosynthesis. These findings establish RC as a safe and effective biomaterial for ethanol‐associated gastropathy and identify G4‐mediated activation of the Pla2g4a‐prostaglandin axis as a key pathway contributing to its gastroprotective effects.

## Graphical abstract

Orally administered recombinant human type III collagen protects the gastric mucosa from ethanol‐induced injury by coordinating redox homeostasis, epithelial barrier repair, and arachidonic acid–prostaglandin metabolism. Gastric digestion releases the pentapeptide GPAEF, which interacts with the Pla2g4a promoter G‐quadruplex in a structure‐dependent manner and modulates its conformation, providing a mechanistic link between collagen digestion and mucosal defense.

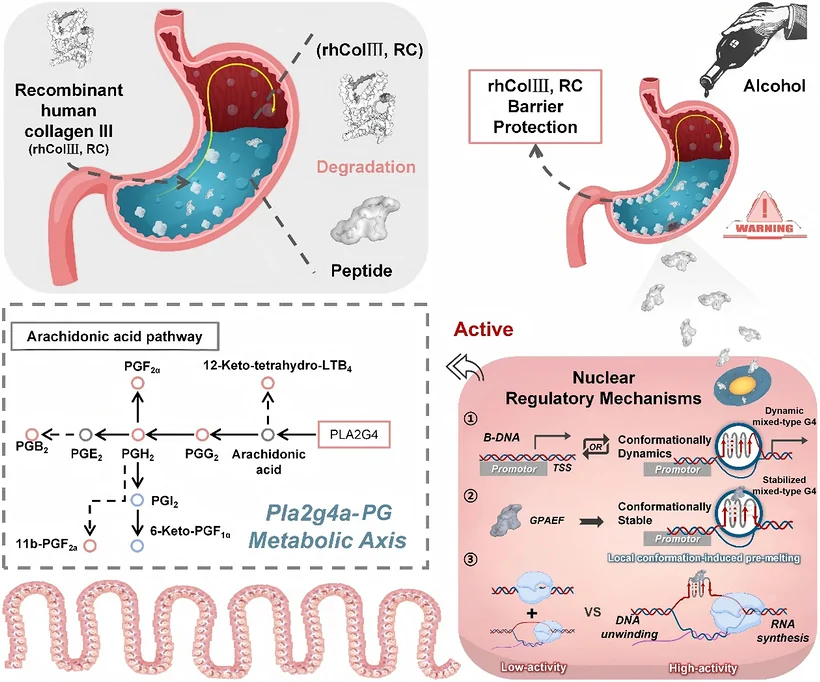

## 1 Introduction

Ethanol‐induced acute gastric mucosal injury (AGMI) is a pathological condition that arises from short‐term excessive intake of high‐concentration ethanol, leading to disruption of the gastric mucosal barrier. The main pathological mechanisms include direct dissolution of the phospholipid bilayer on the mucosal surface, excessive production of reactive oxygen species (ROS) induced by ethanol and its metabolite acetaldehyde, and inflammatory responses that disrupt tight junctions, increase epithelial permeability, and promote inflammatory cell infiltration [1, 2, 3, 4]. Epidemiological data indicate that 15%–35% of short‐term heavy drinkers develop gastric mucosal erosions or hemorrhagic gastritis, and some cases progress to gastric ulcers, perforation, or even cancer [5, 6]. As alcohol use disorders continue to rise globally, alcohol‐related diseases impose a substantial medical and socioeconomic burden, with more than 3 million deaths attributed to ethanol abuse in 2018 alone [6].

Current clinical management of ethanol‐induced AGMI primarily relies on four therapeutic strategies: (1) *topical protective agents*, such as antacids and sucralfate, which neutralize gastric acid and form a physical barrier on the mucosal surface [2, 7]. (2) *H_2_ receptor antagonists*, such as ranitidine, which inhibit acid secretion by blocking H_2_ receptors [8]. (3) *Proton pump inhibitors* (PPIs, including omeprazole and lansoprazole [9]), which specifically inhibit parietal cell H^+^/K^+^‐ATPase activity, thereby blocking the terminal step of acid secretion [9]. (4) *Prostaglandin analogs*, which enhance mucosal defense by increasing blood flow and mucus secretion [10]. However, long‐term use of H_2_ receptor antagonists and PPIs is associated with cardiovascular, hepatic, and renal toxicity and can disrupt of the gut microbiota [11, 12], while the non‐selective pharmacological effects of prostaglandin analogs limit their clinical utility due to systemic adverse reactions [13, 14]. These limitations highlight the need for safer and more mechanistically precise interventions, particularly those based on bioactive molecules with multimodal regulatory potential.

In recent years, bioactive components derived from natural products have attracted increasing attention in the prevention and treatment of gastric mucosal injury [2, 15]. Polysaccharides have been reported to exert mucosal protective effects through immunomodulation [16, 17], phytochemicals are extensively studied for their multi‐target antioxidant properties [18], and bioactive peptides (e.g., wheat peptides [19], fish collagen peptides [20]) have shown dual antioxidative and anti‐inflammatory activities [19, 20, 21]. Nonetheless, this field still faces multiple technical and conceptual bottlenecks. At the raw material level, plant‐ and animal‐derived collagens often exhibit high immunogenicity and disordered fibril assembly; conventional extraction methods fail to yield highly purified, structurally defined collagen fractions, which hampers both clinical translation and mechanistic elucidation. At the mechanistic level, most studies selectively target single pathological links (such as oxidative stress or inflammation) and rarely address how coordinated regulatory networks are remodeled during gastric mucosal injury and repair. Longitudinal analyses of dynamic metabolic responses in gastric tissues after intervention are scarce, and a systematic framework for understanding metabolic reprogramming in AGMI is still lacking. Moreover, current research has predominantly focused on low‐molecular‐weight collagen peptides and their degradation products (e.g., glycyl‐proline fragments [20, 22]), under the assumption that small peptides are more readily absorbed and bioavailable. This emphasis may have led to the relative neglect of mechanistic exploration of macromolecular collagens themselves, despite their potential contribution to structural integrity, toughness, and sustained bioactivity.

Recombinant human type III collagen (rhColIII, RC), a core component of the extracellular matrix, has emerged as a promising biomaterial for tissue repair owing to its excellent biocompatibility, absence of animal‐origin risks, and well‐defined molecular structure [23, 24, 25, 26]. RC can act as a biomimetic scaffold to mediate mechanical signal transduction, guide epithelial cell migration, and modulate cell–matrix interactions; it also engages integrin receptors and downstream signaling pathways such as FAK/RhoA/ROCK, thereby promoting fibroblast activation, extracellular matrix synthesis, and controlled inflammatory responses [25, 27, 28, 29, 30]. These properties provide a strong theoretical basis for considering RC as a candidate biomaterial for gastric mucosal protection. However, existing studies on RC have mainly focused on surface tissues such as skin and cartilage [23, 25]. In contrast, the gastric mucosa is continuously exposed to a uniquely harsh environment characterized by high acidity, dynamic mechanical stress from peristalsis, and complex interactions with luminal metabolites and microbiota. These features suggest that the mechanisms by which RC modulates gastric mucosal repair may differ fundamentally from those in dermal or cartilage tissues and cannot simply be extrapolated from previous work, underscoring a critical and as yet unexplored research gap.

In this study, we systematically investigate the efficacy and mechanism of action of orally administered recombinant human type III collagen (RC) as a gastroprotective biomaterial in ethanol‐induced AGMI. Using GES‐1 human gastric mucosal epithelial cells, we first evaluate the biosafety of RC and define an optimal protective concentration range at the cellular level, laying the groundwork for in vivo studies. We then establish a rat model of ethanol‐induced AGMI and demonstrate that a 30‐day oral pre‐intervention with RC markedly prevents gastric mucosal injury, reducing lesion area and improving histological and biochemical indices compared with animal‐derived collagens.

To elucidate the underlying mechanism, we integrate metabolomic and transcriptomic analyses and show that RC induces a coordinated, tripartite response: maintaining redox homeostasis by reinforcing glutathione‐centered antioxidant pathways, restoring the cytoskeletal and tight junction barrier to preserve mucosal integrity, and rewiring metabolic‐inflammatory networks to favor epithelial regeneration—collectively reflecting an RC‐induced metabolic reprogramming of gastric tissue. Central to these effects is a *Pla2g4a*‐prostaglandin (PG) axis, whereby RC specifically engages the arachidonic acid‐prostaglandin pathway and promotes the synthesis of prostaglandin‐related protective mediators [31, 32, 33]. We identify a gastric‐digested pentapeptide, GPAEF, derived from RC that selectively recognizes and stabilizes the *Pla2g4a* promoter G‐quadruplex (G4), promotes remodeling of its mixed‐type conformational ensemble, and stabilizes the peptide‐G4 complex; this conformational response may contribute to enhanced *Pla2g4a* transcription and downstream PG biosynthesis. Taken together, our findings support a multiscale mechanistic model linking oral RC administration and the in vivo gastric generation of GPAEF, the experimentally observed in vitro interaction between GPAEF and a gene‐regulatory *Pla2g4a* G4 element, and RC‐associated activation of the *Pla2g4a*‐prostaglandin axis, thereby providing a plausible mechanistic link to gastric mucosal protection. This work establishes RC as a safe and effective oral biomaterial for ethanol‐associated gastropathy and provides a mechanistic framework for G4‐mediated epigenetic‐metabolic regulation in gastric mucosal defense.

## 2 Materials and Methods

### 2.1 Materials and Reagents

GES‐1 cells were obtained from Baidi Biotechnology Co., Ltd (Zhejiang, China). Recombinant human type III collagen (rhColIII, RC) was supplied by Jiangsu Chuangjian Medical Technology Co., Ltd. RPMI‐1640 medium, Fetal bovine serum (FBS, Gibco), Trypsin‐EDTA (Gibco), a penicillin‐streptomycin‐neomycin (PSN) antibiotic mixture, and PBS buffer (pH 7.2) were purchased from Sigma‐Aldrich Inc. (USA). Fluorescent probe 2,7‐dichlorofluorescein diacetate (DCFH‐DA) assay kit, mitochondrial Membrane Potential Detection Kit (JC‐1), Cell Counting Assay (CCK‐8), and BCA protein assay kit were purchased from Beyotime Biotechnology Co., Ltd. (Shanghai, China). Analytical pure anhydrous ethanol was purchased from Shanghai Guoyao. Reduced glutathione (GSH) assay kit and Superoxide Dismutase (SOD) assay kit (WST‐1 method) were purchased from Nanjing Jiancheng Bioengineering Institute.

### 2.2 Cell Experiments

#### 2.2.1 Cell Culture and Treatment

GES‐1 cells were cultured in 75 cm^2^ flasks with RPMI‐1640 medium supplemented with 10% (v/v) FBS and a penicillin‐streptomycin‐neomycin (PSN) antibiotic mixture. The cultures were incubated at 37°C in a 5% CO_2_ atmosphere. Cells were washed with PBS (pH 7.2) and detached by using Trypsin‐EDTA.

To explore the method of establishing ethanol‐induced GES‐1 cell damage model, cells were exposed to ethanol at various concentrations: 5%, 6%, 7%, 8%, 9%, and 10% for 1 h to determine cell viability. The ethanol concentration corresponding to a cell viability of approximately 60% was selected as the optimal modeling concentration. GES‐1 cells were pretreated with various concentrations of RC for 24 h, following ethanol exposure at the selected concentration for 1 h. The protective effects of RC on cell viability, oxidative stress, and inflammation were subsequently evaluated.

#### 2.2.2 Cell Viability and Cytotoxicity Analysis

The effects of RC on the viability and cytotoxicity of GES‐1 cells were evaluated using a Cell Counting Kit‐8 (CCK‐8) assay. The GES‐1 cells were pre‐incubated in a 96‐well plate (10 000 cells/100 µL/well) in RPMI‐1640 medium with 10% calf serum. After cell proliferation to 70%–80% confluency, different concentrations of RC was added to GES‐1 cells and incubated at 37°C in 5% CO2. After treatment for 24 h, the RPMI‐1640 medium was removed, and the plates were washed twice with RPMI‐1640 medium. Next, 100 µL of fresh RPMI‐1640 medium (10% calf serum) and 10 µL of the CCK‐8 solution were added, sequentially. After incubation for 2 h, the absorbance was measured at 450 nm using an enzyme reader (Tecan Infinite 200 PRO, Austria). The relative cell viability was determined as cell survival percentage in comparison to cells that were only treated with complete RPMI‐1640 medium (controls).

#### 2.2.3 Scratch Assay

The GES‐1 cells were cultured in 6‐well plates (5 × 10^5^ cells/well) until they form a confluent layer. A scratch was made across the cell monolayer using a 100 µL sterile pipette tip. The medium was removed, and the cells were washed with PBS to eliminate any remaining detached cells. Subsequently, various concentrations of RC in RPMI‐1640 medium supplemented with 1% FBS were added to each well. The cell‐free areas were photographed and quantified at 0 and 48 h using a Leica DMi8 manual inverted fluorescence microscope (Leica Microsystems). All experiments were conducted in triplicate. ImageJ software was used to analyze the scratch area.

#### 2.2.4 Intracellular ROS Generation Assay

Intracellular ROS production in GES‐1 cells was assessed using 2,7‐dichlorofluorescein diacetate (DCFH‐DA). Following treatment with various concentrations of RC and 6% anhydrous ethanol, the GES‐1 cells were incubated with 10 µM DCFH‐DA at 37°C for 30 min under light protection. ROS levels were quantified using a fluorescent enzyme reader, and fluorescence images were captured using a Zeiss LSM900 laser scanning confocal microscope (Zeiss, Inc., Germany).

#### 2.2.5 Mitochondrial Membrane Potential Assay

Mitochondrial Membrane Potential Detection Kit (JC‐1) was used to assess changes in mitochondrial membrane potential caused by RC treatment in ethanol‐damaged GES‐1 cells. JC‐1 monomer fluorescence (green) was observed at excitation/emission (Ex/Em) 515 nm/529 nm, and JC‐1 polymer fluorescences (red) were detected at excitation/emission (Ex/Em) 585/590. Changes in the potential of the mitochondrial membrane were detected by the shift in fluorescence color. Treated GES‐1 cells in confocal dishes were incubated with JC‐1 reagent (10 µM, 1 mL/well) for 20 min at 37°C protected from light and then imaged with confocal microscopy. The ratio of red to green fluorescence was utilized for determining changes in the mitochondrial membrane potential. A fluorescent enzyme reader was also used to quantify these changes in a 96‐well plate.

#### 2.2.6 GSH and SOD Determination

The GSH content and SOD activity were determined separately using commercial kits according to the instructions (Jiancheng, Nanjing, China). The protein level of GES‐1 cells was determined using BCA Protein Assay Kit (Beyotime, Beijing, China) to normalize the GSH and SOD activity levels.

### 2.3 Animal Experiments

#### 2.3.1 Animals and Grouping

Male Wister rats (SPF grade), weighing 180–220 g were provided by Beijing Huafukang Biotechnology Co., Ltd. The rats were reared in a room with controllable temperature and humidity (22°C–23°C and 46%–63%, respectively) and were allowed ad libitum access to drinking water and chow. After one week of acclimation, the rats were randomly grouped. To investigate the protection effects of recombinant collagenon (RC) on ethanol‐induced gastric mucosal injury in rats, six groups (10 rats per group) were set up for comparative analysis: normal (negative), model (positive), RC low‐dose group, RC medium‐dose group, RC high‐dose group and commercial fish collagen group (same dose as RC High‐dose). Commercial fish collagen was included as a category‐matched material comparator to assess the relative preventive performance of RC against a conventional animal‐derived collagen preparation under the same dosing regimen. All animal experiments were approved by the Animal Care and Use Committee of China Agricultural University (AW80013202‐5‐3).

#### 2.3.2 Ethanol‐Induced Acute Gastric Injury in Rats

In animal experiments, according to China Test Methods for Auxiliary Protection of Gastric Mucosal Damage (2012) and China Technical Guidelines for Toxicological Testing and Evaluation of Health Foods and Their Raw Materials (2020) and a previously reported method by Lu et al. (Lu, Kong et al. 2022). Preliminary experiments were conducted to determine the appropriate doses. The dose conversion and experimental grouping are summarized in Tables S1 and S2, respectively. The rats were gavaged with the tested substance (RC low‐dose group, RC medium‐dose group, RC high‐dose group) at different doses of 0.18 g/kg·BW、0.9 g/kg·BW、4.5 g/kg·BW, while the commercial fish collagen group received fish collagen at 4.5 g/kg BW. The normal and model groups received an identical amount of distilled water. This gavage was once a day for thirty‐two consecutive days. Food consumption were recorded daily, body weight were recorded twice a week. On day 30, feces were collected for 16S rRNA sequencing, metabolomics analysis, and combined microbial and metabolomic analysis. On day 31, the rats fasted with free access to water. On day 32, after giving the tested substance, the rats were given alcohol at a dose of 1mL/kg BW to induce gastric mucosal injury, and the normal group was given an equal volume of distilled water. One hour later, the rats were sacrificed. The blood, stomachs, and other organs were collected for further analysis.

#### 2.3.3 Macroscopic Assessment of Gastric Mucosal Injury

After euthanasia, each stomach was excised, opened along the greater curvature, gently rinsed with physiological saline, and fully spread to expose the entire gastric mucosal surface. The complete exposed mucosal surface was photographed and examined for tissue integrity, surface smoothness, color, bleeding, erosion, and other abnormalities. Macroscopic gastric mucosal injury was scored according to the criteria presented in Table S4. The number of bleeding points and the length and width of bleeding bands were recorded, and the injury incidence rate were calculated from the macroscopic scores. The damaged gastric mucosal area was further quantified with the bright‐red regions identified as damaged areas. The percentage of gastric mucosal injury was calculated as follows:

$$\mathrm{Injury}\;\mathrm{area}\;\mathrm{percentage}\;\left(\%\right)=\frac{Area\;gasrtic\;injury}{Area\;gastric\;tissue}\;\times \;100\%$$

#### 2.3.4 Histopathological Evaluations

For histopathological examination, the region showing the most severe macroscopic mucosal injury in each animal was collected according to the standardized sampling procedure specified in the above‐mentioned guidelines. The tissue was fixed in 10% neutral buffered formalin, routinely dehydrated, paraffin‐embedded, sectioned, and stained with hematoxylin and eosin (H&E). A transverse section containing the complete mucosal layer was selected for microscopic examination. Histopathological injury, including mucosal congestion, hemorrhage, and epithelial degeneration or necrosis, was scored according to the criteria presented in Table S5. Low‐magnification images of the analyzed tissue sections were acquired, and the explicitly boxed regions were further imaged at higher magnification. The damaged tissue area was quantified by segmenting normal mucosa, damaged regions, and muscle or other tissues.

#### 2.3.5 Biochemical Assessment of Gastric Tissue Redox Status

To directly evaluate gastric tissue redox homeostasis, gastric tissue samples from the Normal, Model, RC High‐dose, and commercial fish collagen groups were subjected to biochemical analysis. Total glutathione and oxidized glutathione (GSSG) were determined using a GSH and GSSG Assay Kit (S0053, Beyotime Biotechnology, Shanghai, China) according to the manufacturer's instructions. Tissue samples were deproteinized before analysis. Reduced GSH was calculated as total glutathione minus two times the GSSG content, and the GSH/GSSG ratio was subsequently calculated. Glutathione contents were normalized to tissue weight.

Lipid peroxidation was assessed by measuring malondialdehyde (MDA) using a Lipid Peroxidation MDA Assay Kit (S0131S, Beyotime Biotechnology). Gastric tissue homogenates were reacted with thiobarbituric acid, and absorbance was measured at 532 nm. MDA content was normalized to total protein concentration determined by the BCA assay and expressed as nmol/mg protein.

Glutathione peroxidase (GPx) activity was determined using a Cellular Glutathione Peroxidase Assay Kit with NADPH (S0056, Beyotime Biotechnology). The decrease in NADPH absorbance at 340 nm was monitored kinetically at 25°C, and specific GPx activity was expressed as ΔOD340/min/mg protein after normalization to total protein concentration. Representative standard curves and kinetic profiles used for assay validation are provided in Figure S6.

#### 2.3.6 Targeted Quantification of Prostaglandin Metabolites in Gastric Tissue

Gastric tissue samples from the Normal, Model, RC High‐dose, and commercial fish collagen groups were used for targeted quantification of prostaglandin E2 (PGE2) and 6‐keto‐prostaglandin F1α (6‐keto‐PGF1α). Gastric tissues were homogenized at a 1:9 (w/v) ratio in ice‐cold PBS supplemented with protease and phosphatase inhibitor cocktails, followed by centrifugation to collect the tissue homogenate supernatants.

PGE2 and 6‐keto‐PGF1α were quantified using commercial competitive ELISA kits (Rat PGE2 ELISA Kit, catalog no. QTJX30697; Rat 6‐keto‐PGF1α ELISA Kit, catalog no. QTJX30025) according to the manufacturers’ instructions. Briefly, standards or tissue homogenate samples were added to antibody‐coated microplates together with the corresponding HRP‐labeled antigens. After incubation and washing, substrate solutions were added for color development, followed by reaction termination. Absorbance was measured at 450 nm using a Spark multimode microplate reader (Tecan, Männedorf, Switzerland). Analyte concentrations were calculated from the corresponding calibration curves using logit‐log regression, in which the logit‐transformed absorbance response was fitted against the natural logarithm of analyte concentration. The PGE2 and 6‐keto‐PGF1α calibration curves are provided in Figure S7A,B. Total protein concentrations were determined using the BCA assay, and PGE2 and 6‐keto‐PGF1α contents were normalized to total protein and expressed as pg/mg protein.

#### 2.3.7 Determination of Total cPLA2 and p‐cPLA2 (Ser505) Levels and cPLA2 Enzymatic Activity in Gastric Tissue

Gastric tissue samples from the Normal, Model, RC High‐dose, and commercial fish collagen groups were used to determine total cPLA2 protein abundance, phosphorylated cPLA2 (p‐cPLA2), and cPLA2 enzymatic activity, with four independent biological replicates per group.

For total cPLA2 and p‐cPLA2 measurements, gastric tissues were homogenized at a 1:9 (w/v) ratio in ice‐cold PBS supplemented with protease and phosphatase inhibitor cocktails. The homogenates were centrifuged at 5000 × g for 10 min at 4°C, and the resulting supernatants were collected. Total cPLA2 and p‐cPLA2 (Ser505) levels were quantified using commercial rat cPLA2 and p‐cPLA2 ELISA kits (catalog nos. SU‐BN30234 and SU‐BN205195, respectively) according to the manufacturers’ instructions. Absorbance was measured at 450 nm using a Spark multimode microplate reader (Tecan, Männedorf, Switzerland), and protein concentrations were calculated from the corresponding calibration curves. Total cPLA2 and p‐cPLA2 levels were normalized to total protein concentration determined using the BCA assay and expressed as pg/mg protein. The p‐cPLA2/cPLA2 ratio was calculated to evaluate phosphorylation‐associated cPLA2 activation relative to total cPLA2 protein abundance. The corresponding ELISA calibration curves are provided in Figure S8.

For cPLA2 enzymatic activity, separate gastric tissue aliquots were homogenized in 5–10 mL/g tissue of ice‐cold 50 mM HEPES buffer (pH 7.4) containing 1 mM EDTA. After centrifugation at 10 000 × g for 15 min at 4°C, the supernatants were collected and analyzed using a cPLA2 Assay Kit (Item No. 765021, Cayman Chemical, Ann Arbor, MI, USA). To minimize interference from calcium‐independent phospholipase A2 (iPLA2), tissue homogenate supernatants were preincubated with Bromoenol Lactone at a final concentration of 5 µm for 15 min at 25°C. cPLA2 activity was subsequently measured using arachidonoyl thio‐PC as the substrate, with released thiols detected using DTNB at 414 nm. Enzymatic activity was calculated according to the manufacturer's instructions, normalized to total protein concentration determined using the BCA assay, and expressed as nmol/min/mg protein.

#### 2.3.8 In Vivo Toxicity Studies

For in vivo toxicity studies, organs, including heart, liver, spleen, lung, kidney, brain, adrenal gland, testis, thymus, duodenum, and epididymis were harvested, fixed with 10% formalin, and then embedded in paraffin for H&E staining.

#### 2.3.9 Hematology Analysis

Venous blood samples were collected aseptically from the jugular vein with disposable vacutainers. All samples were collected before killing rats. Whole blood was used for routine test and biochemistry using an automated hematology analyzer (Bio Line, BL‐6500, China): hemoglobin concentration (Hb), red blood cell count (RCCs), PCV, white blood cell count (WBCs), platelet count (PLTs), total cholesterol (chol), HDL cholesterol (HDL), LDL cholesterol (LDL), and so on.

#### 2.3.10 Quantitative Real‐Time PCR Analysis

Total RNA was extracted from the gastric tissues using Trizol reagent (Thermo Fisher Scientific). Equal amounts of RNA were used to synthesize the cDNA using FastKing gDNA Dispelling RT Supermix (Tiangen Biotech, Beijing, China). The real‐time PCR analysis for each samples from all four experimental groups and the corresponding ACTB (β‐Actin was used as the reference gene with FP (5’‐GTCCACCCGCGAGTACAAC‐3’) and RP (5’‐TATCGTCATCCATGGCGAACTGG‐3’)) reference reactions were analyzed together. Gene expression was quantified by SYBR Green‐based quantitative real‐time PCR. The mean Cq value was used for calculation. Relative gene expression was calculated using the 2^−ΔΔCq^ method, where ΔCq = Cq__target_ – Cq_ACTB_ and the mean ΔCq of the Normal group was used as the calibrator. Reactions were included when they exhibited a typical sigmoidal amplification curve and a single predominant melting peak. The primer pairs were adopted from previously published rat RT‐qPCR studies. The primer sequences and original references, qPCR program, raw cycle‐by‐cycle fluorescence data, amplification and melting curves, raw Cq values, and normalization calculations are provided in Data S1.

#### 2.3.11 Gastric Lavage Collection and Targeted LC‐MS/MS Analysis of GPAEF

An independent time‐course experiment was performed using male Wistar rats (specific pathogen‐free grade), weighing 280–320 g, to evaluate the gastric occurrence of GPAEF following oral RC administration. After fasting for 16 h with free access to water, the animals received a single oral gavage of RC at 4.5 g/kg body weight. Animals were euthanized at 0, 15, 30, 120, and 240 min after administration (n = 3 animals per time point). The stomach was excised and opened, and the residual gastric contents together with mucus adhering to the gastric mucosal surface were collected by thoroughly washing the stomach with a total of 8 mL PBS. The resulting gastric mucosal lavage fluid was retained for targeted LC‐MS/MS analysis. All animal experiments were approved by the Animal Care and Use Committee of China Agricultural University (AW01606202‐5‐01).

For sample preparation, 200 µL of gastric lavage fluid was transferred to a 1.5‐mL centrifuge tube, and acetonitrile was added to a final volume of 1 mL. After vortex mixing for 5 min, the samples were centrifuged at 12 000 rpm for 5 min. The supernatant was passed through a 0.22‐µm organic‐solvent‐compatible membrane filter, and 5 µL was injected for analysis. Chromatographic separation was performed on an Agilent 1290 liquid chromatography system equipped with a C18 column at a flow rate of 0.3 mL/min, coupled to an Agilent 6460B triple‐quadrupole mass spectrometer operated with an Agilent Jet Stream ESI source in positive‐ion multiple‐reaction‐monitoring (MRM) mode. GPAEF was monitored using m/z 520.2→155.0 as the quantifier transition and m/z 520.2→355.0 and 520.2→166.1 as qualifier transitions. The corresponding collision energies were 32, 16, and 36 V, respectively, with a fragmentor voltage of 162 V. Source parameters were as follows: gas temperature, 300°C; gas flow, 5 L/min; nebulizer pressure, 45 psi; sheath‐gas temperature, 250°C; sheath‐gas flow, 11 L/min; and capillary voltage, 3500 V.

Quantification was performed using matrix‐matched external calibration standards prepared in blank gastric lavage matrix. Nine nominal levels (0.1, 0.5, 1, 2, 5, 10, 20, 50, and 100 ng/mL) were initially evaluated. Positive identification required retention‐time agreement with the GPAEF standard and concordant signals from the quantifier and qualifier transitions. Signals below the response of the lowest retained calibration standard or failing qualifier‐ion criteria were reported as not detected (ND). Detailed calibration performance and individual analytical results are provided in Tables S11 and S12.

### 2.4 Combined Multi‐Omics Analysis

#### 2.4.1 16S rRNA Sequencing

After collecting the feces, immediately freeze them in liquid nitrogen and stored at −80°C for subsequent DNA extraction and microbial analysis. FP(5’‐ACTCCTACGGGAGGCAGCA‐3’) and RP (5’‐GGACTACHVGGGTWTCTAAT‐3’) were used for amplification. The 16S rRNA gene sequences were analyzed using QIIME platform scripts (www.qiime.org). The reference sequences were selected into operational taxonomic units (OTUs) by clustering 97% sequence similarity. The ribosomal database project classifier was applied to systematically classify OTU sequences with reference to the Silva database.

#### 2.4.2 Analyses of Metabolites of Gastric Mucosa

Liquid chromatography‐mass spectrometry (LC‒MS) analysis was performed to identify the gastric mucosa and feces. LC analysis was performed on a Vanquish UHPLC System (Thermo Fisher Scientific, USA). Chromatography was carried out with an ACQUITY UPLC HSS T3 (2.1 × 100 mm, 1.8 µm) (Waters, Milford, MA, USA). The column maintained at 40°C. The flow rate and injection volume were set at 0.3 mL/min and 2 µL, respectively. For LC‐ESI (+)‐MS analysis, the mobile phases consisted of (B2) 0.1% formic acid in acetonitrile (v/v) and (A2) 0.1% formic acid in water (v/v). Separation was conducted under the following gradient: 0–1 min, 8% B2;1–8 min, 8%–98% B2;8–10 min, 98% B2;10–10.1 min, 98%–8% B2;10.1–12 min, 8% B2. For LC‐ESI (‐)‐MS analysis, the analytes were carried out with (B3) acetonitrile and (A3) ammonium formate (5 mM). Separation was conducted under the following gradient: 0–1 min, 8% B3;1–8 min, 8%–98% B3;8–10 min, 98% B3;10–10.1 min, 98%–8% B3.

Mass spectrometric detection of metabolites was performed on an ORCitrap Exploris 120 (Thermo Fisher Scientific, USA) with an ESI ion source. Simultaneous MS1 and MS/MS (full MS‐ddMS2 mode, data‐dependent MS/MS) acquisition was used. The parameters were as follows: sheath gas pressure, 40 a RC; aux gas flow, 10 a RC; spray voltage, 3.50 kV and ‐2.50 kV for ESI( +) and ESI(‐), respectively; capillary temperature, 325°C; MS1 range, m/z 100–1000; MS1 resolving power, 60 000 FWHM; number of data‐dependent scans per cycle, 4; MS/MS resolving power, 15 000 FWHM; normalized collision energy, 30%; and dynamic exclusion time, automatic.

#### 2.4.3 Combined Analysis of Microbial and Metabolomic Data

Differential microbiota analysis and metabolites of feces was conducted by assessing 16SrRNA and LC‐MS. Correlation analyses of differential microbiota and metabolites were performed using R packages.

#### 2.4.4 Integrated Analysis of Transcriptomic and Metabolomic Data

Transcriptomic and metabolomic datasets were generated from separate gastric tissue pieces collected from the same stomach and were matched by animal identity before integration. The normalized expression matrix of differentially expressed genes and the normalized abundance matrix of differential metabolites were aligned across the matched samples, and the Model versus Normal and RC‐high versus Model comparisons were analyzed separately. Gene co‐expression modules were identified using the WGCNA package in R. A weighted co‐expression network was constructed according to similarities in gene‐expression patterns, and modules were defined by hierarchical clustering based on topological overlap. Each module was summarized by its module eigengene, which was subsequently correlated with metabolite abundance; the resulting correlation coefficients and P values were visualized as module‐metabolite correlation heatmaps.

For focused pairwise analysis, representative genes and metabolites were selected according to the differential analyses, pathway‐enrichment results, and their relevance to the major lipid‐ and amino acid‐metabolic pathways. Pairwise Kendall's τ coefficients and corresponding P values were calculated using the linkET package in R and visualized as butterfly and bipartite correlation plots. Positive and negative correlations were distinguished by color, whereas line width represented the P‐value category. Finally, pathway annotations were integrated to construct a gene‐metabolite association network in Cytoscape. Metabolites and pathway‐associated genes were represented by red octagons and blue triangles, respectively, and gray edges denoted pathway‐based associations.

### 2.5 In Vitro Validation Experiment

#### 2.5.1 Structure Prediction

For structure predictions, we utilized the AF3 server (https://alphafoldserver.com/). GPAEF sequences were submitted. Then molecules were in total predicted. The random seed was set to its default state, and the top‐ranked predicted structure was selected for further comparative analysis.

#### 2.5.2 Oligonucleotide Design and Circular Dichroism Spectroscopy

The wild‐type *Pla2g4a* promoter G‐quadruplex‐forming sequence (*Pla2g4a*‐G4) and two G4‐disrupting mutant sequences, designated G4‐mutA and G4‐mutT, were synthesized. The mutant sequences contained substitutions within the guanine tracts to disrupt or markedly weaken the G4‐forming capacity of the wild‐type sequence. The complete wild‐type and mutant oligonucleotide sequences and the corresponding mutation sites are provided in Table S10.

GPAEF was prioritized from the gastric digestion peptidomic dataset because it exhibited the highest identification confidence and relative abundance among the detected pentapeptides. Four additional representative high‐abundance pentapeptides, KGETG, PGPAE, PGAPG, and AGQPG, were selected from the same dataset for comparative analysis. Pentapeptides of the same chain length were used to reduce potential differences arising from peptide length and molecular size.

Before structural analysis, oligonucleotides were heated at 95°C for 5 min and slowly cooled to room temperature to allow folding. Circular dichroism (CD) spectra were recorded using 10 µM oligonucleotide in 5 mM Tris‐HCl buffer (pH 7.4), either alone or supplemented with 50 mM KCl or 50 mM NaCl. For peptide‐comparison experiments, *Pla2g4a*‐G4 was preincubated with each peptide at 0.1 mg/mL for 30 min at room temperature under identical conditions. Wild‐type and mutant sequences were analyzed in both Tris‐KCl and Tris‐HCl buffer systems.

Spectra were collected from 200 to 320 nm at 25°C using a Chirascan Plus circular dichroism spectrometer (Applied Photophysics Ltd., Leatherhead, UK) equipped with a 0.1 cm path‐length quartz cuvette, with a bandwidth of 1.0 nm and an integration time of 0.5 s per point. Three consecutive scans were averaged for each sample, and all spectra were baseline‐corrected using the corresponding solvent blank.

The intrinsic CD spectra of the five free pentapeptides were also recorded to evaluate their individual spectral contributions. GPAEF was further analyzed at 0.1, 0.2, 0.5, and 1.0 mg/mL. Because increasing peptide concentrations caused elevated high‐tension voltage in the low‐wavelength region and reduced the valid scanning range, 0.1 mg/mL was selected for subsequent peptide‐G4 interaction experiments. The corresponding peptide characterization and concentration‐optimization data are provided in Figure S13.

#### 2.5.3 Thioflavin T Fluorescence and Binding‐Affinity Assays

Thioflavin T (ThT) fluorescence assays were performed using 2 µM ThT in 5 mM Tris‐HCl buffer (pH 7.4), either alone or supplemented with 50 mM KCl. Wild‐type *Pla2g4a*‐G4, G4‐mutA, and G4‐mutT were used at a final concentration of 1 µm. The oligonucleotides were pre‐annealed by heating at 95°C for 5 min followed by slow cooling to room temperature.

For peptide‐comparison experiments, each of the five pentapeptides was added at 0.1 mg/mL and incubated with the preformed ThT‐G4 complex for 20 min at 25°C under light‐protected conditions. Fluorescence emission spectra were recorded from 468 to 600 nm with an Agilent fluorescence spectrophotometer (Agilent Technologies, Santa Clara, CA, USA), using an excitation wavelength of 442 nm and excitation/emission slit widths of 10/10 nm. The change in ThT fluorescence intensity was calculated as ΔI = I_before peptide addition − I_after peptide addition at the fluorescence maximum.

To assess the dependence of the GPAEF response on an intact G4 structure, complete ThT emission spectra of the wild‐type and mutant sequences were recorded before and after GPAEF addition in both Tris‐KCl and Tris‐HCl buffer systems.

Apparent dissociation constants for the interactions of *Pla2g4a*‐G4 with GPAEF and ThT were determined from fluorescence titration data by nonlinear regression using the binding model described for the original assay.

#### 2.5.4 Static Molecular Docking

The molecular docking procedure was performed as follows: First, the *Pla2g4a* G4‐forming DNA sequence (5′‐GGGATGAGGGGCAGGGGGAGGG‐3′) was submitted to the 3D‐NuS nucleic acid structure prediction server for three‐dimensional G4 modeling. Based on the experimentally observed CD spectral characteristics, both parallel and hybrid/mixed (Mix) G4 topologies were constructed. For each topology, the model was defined as a three‐G‐tetrad structure, and the intervening loop sequences were entered according to the native *Pla2g4a* sequence. The server then generated the corresponding three‐dimensional G4 structures, which were subjected to energy minimization before subsequent molecular docking. The resulting parallel and hybrid/mixed G4 models were then imported into Discovery Studio 2021 (Dassault Systèmes) and subjected to energy minimization before molecular docking. The energy‐minimized G4 models and GPAEF ligand structure were submitted to the HDOCK server (http://hdock.phys.hust.edu.cn/) for rigid‐body docking simulations, using default parameters with 100 generated poses. The top‐scoring complexes were then refined through energy minimization in Discovery Studio 2021 (Dassault Systèmes). Final binding poses were evaluated through: (1) Visual inspection of interaction patterns (hydrogen bonds, π‐stacking, and so on), (2) Binding energy calculations (MM‐PBSA), and (3) Cluster analysis of docking conformations, etc.

#### 2.5.5 Molecular Docking and Molecular Dynamics Simulation

Molecular docking combined with molecular dynamics (MD) simulations was employed to investigate the interactions between the DNA aptamer and the pentapeptide. The initial binding conformation of the DNA aptamer‐pentapeptide complex was obtained using AutoDock, and the docking pose with favorable binding energy and reasonable geometry was selected as the starting structure for subsequent simulations. All MD simulations were performed using the GROMACS software package (version 2024.6) with the AMBER99SB‐ILDN force field. The complex was placed in a cubic simulation box and solvated with the TIP3P explicit water model, followed by the addition of appropriate numbers of K^+^/Na^+^ and Cl^−^ ions to neutralize the overall system charge. The system was first subjected to energy minimization to remove unfavorable steric contacts, followed by equilibration to allow the system to reach a stable state. After equilibration, an unrestrained production MD simulation was carried out for 100 ns under periodic boundary conditions, with a time step of 2 fs. The resulting trajectories were corrected for periodic boundary conditions and used for further analysis. The structural stability and dynamic behavior of the complex were evaluated by calculating the root‐mean‐square deviation (RMSD), root‐mean‐square fluctuation (RMSF), radius of gyration (RoG), and the number of hydrogen bonds. In addition, the binding free energy between the DNA aptamer and the pentapeptide was estimated using the MM/GBSA method.

### 2.6 Statistical Analysis

Differences between the two groups were compared using Student's t‐test. The data from 3 or more groups were evaluated by one‐way analysis of variance (ANOVA). Significance was considered at *P* < 0.05, and the data are expressed as the mean ± standard error of the mean (SEM).

## 3 Results

### 3.1 Safety Evaluation and Optimal Dosage Determination of RC for Gastric Protection Against Acute Ethanol Injury

**FIGURE 1:**
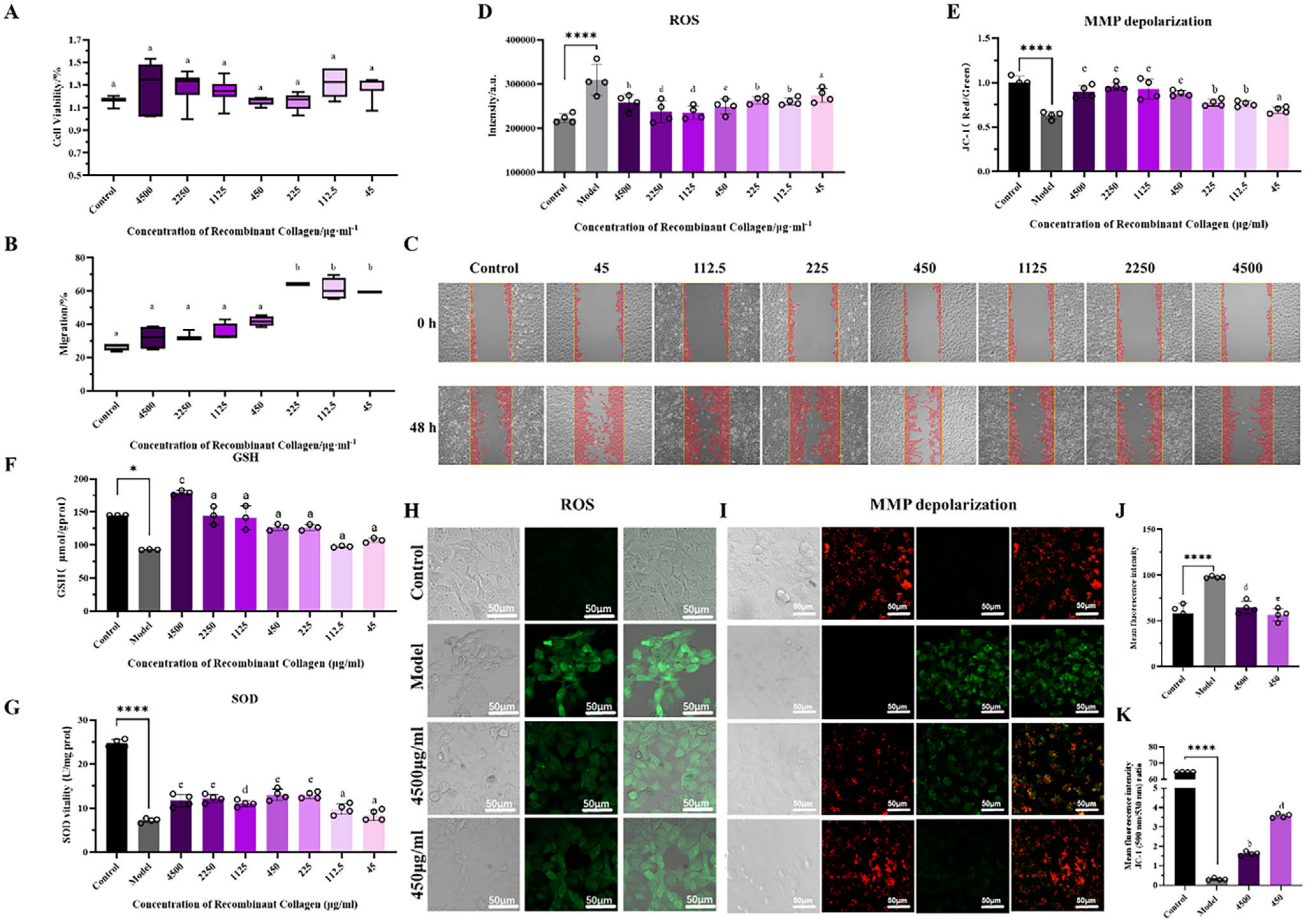
The protective effect on GES‐1 cells of RC: Assessment of dosage safety and repair of acute ethanol‐induced injury. (A) Dose‐dependent cytotoxicity of RC in GES‐1 cells (CCK8). (B) The migrated area ratio of (C) RC‐enhanced cell migration post‐ethanol injury (Scratch assay, 0/48h) (Images were captured with a 20× objective lens of the microscope). RC‐mediated dose‐dependent amelioration of ethanol‐induced (D) ROS accumulation and (E) mitochondrial membrane potential (ΔΨm) collapse (24h). Effect of RC at different doses on intracellular (F)GSH content and (G) SOD Activity in GES‐1 Cells. Confocal imaging of (H) ROS accumulation post‐ethanol injury, and (I)ΔΨm dynamics (JC‐1 staining). Quantitative analysis of (J) ROS fluorescence intensity, and (K) JC‐1 red/green ratio quantification.

Material safety assessment serves as the foundation for efficacy studies. Through infrared spectroscopy comparison, it was found that the characteristic absorption peaks of RC's collagen components exhibited significant overlap with those of porcine type III collagen, which have a well‐established history of safe consumption (Figure S1), indicating their structural similarity and potential edible safety. Next, based on the suitable solubility range of RC, concentration gradients ranging from 0 to 4500 µg/mL were set. *CCK‐8* assays demonstrated that RC at all tested concentrations showed no significant impact on the viability of gastric epithelial cells (GES‐1) (Figure 1A), as well as all doses could promoted cell migration (Figure 1B). Notably, low‐dose groups (<450 µg/mL) exhibited statistically significant proliferation‐promoting effects in scratch assays (Figure 1C).

Then, an acute injury model was established using 6% ethanol to suppress cell viability to 60% (Figure S2). By assessing the ROS levels and mitochondrial membrane potential (JC‐1 Red/Green ratio), the medium‐high RC doses (450–2250 µg/mL) demonstrated both the most potent antioxidant effects and superior ΔΨm restoration capacity (Figure 1D,E). Especially, these effects showed a clear dose‐dependent negative correlation (Figure S3), suggesting RC protects against ethanol‐induced damage through mitochondrial function preservation.

Furthermore, the analysis of protein‐level antioxidant markers revealed that intracellular reduced glutathione (GSH) levels exhibited a strict dose‐dependent recovery trend with increasing RC treatment concentrations. The highest dose group (4500 µg/mL) showed GSH content not only significantly higher than the alcohol‐injured model group (*p* < 0.01) but also exceeding the normal control group by approximately 15% (*p* < 0.05) (Figure 1F), demonstrating robust antioxidant reserve capacity. In contrast, superoxide dismutase (SOD) activity peaked in the moderate‐dose 450 µg/mL group, increasing nearly 40% compared to the model group and approaching normal physiological levels most closely. Although the high‐dose group also exhibited significantly enhanced SOD activity versus the injured group, it remained below the normal control benchmark (Figure 1G). This differential dose response suggests that RC modulates the antioxidant system through distinct mechanisms: high concentrations directly augment GSH‐dependent radical scavenging capacity, while moderate concentrations preferentially restore homeostasis of endogenous antioxidant enzymes like SOD. However, the above results did not show a clear consistency in the selection of the optimal dosage. It can be seen that the low‐dose group exhibited better migration‐promoting effects under steady‐state conditions, whereas medium‐high doses groups showed antioxidant advantages under the state of acute ethanol injury. This suggests that cells require distinct optimal dosage strategies under physiological versus pathological conditions. That is the low dose may stimulate motility and tissue coverage, but this dosage remains insufficient to initiate post‐injury repair processes. While, although the medium‐high doses may not significantly promote proliferation which may depending on the concentration or saturation of collagen itself, it may better meet the effect of resisting ethanol toxicity by activating the anti‐inflammatory antioxidant pathway.

To better clarify the dynamic effect of RC in improving acute ethanol‐induced injury and repair, we extended observation through acute ethanol injury phases (24h) to the early recovery phases (48h) after injury was extended. It could be seen that the 450 µg/mL mid‐dose group exhibited near‐normal spindle morphology, whereas the model group showed pseudopod extension (purple arrows, Figure S4) and cell shrinkage. Moreover, this group showed significantly better performance than the 4500 µg/mL high‐dose group in both ROS clearance efficiency (Figure 1H, J) and ΔΨm restoration (Figure 1I,K) compared to 24h results, indicating its superior stress resistance. Therefore, considering the dose‐effect relationship and potential metabolic burden, we selected 450 µg/mL as the optimal in vitro concentration. Based on the differences in bioavailability between cells and animals, as well as the body surface area conversion, the optimal dose for animal experiments was determined to be 4.5 g/kg (a 10‐fold dosage relationship, see Table S1 for conversion).

### 3.2 Recombinant Collagen‐Mediated the Prevention of Ethanol‐Induced Acute Gastric Mucosa Injury

**FIGURE 2:**
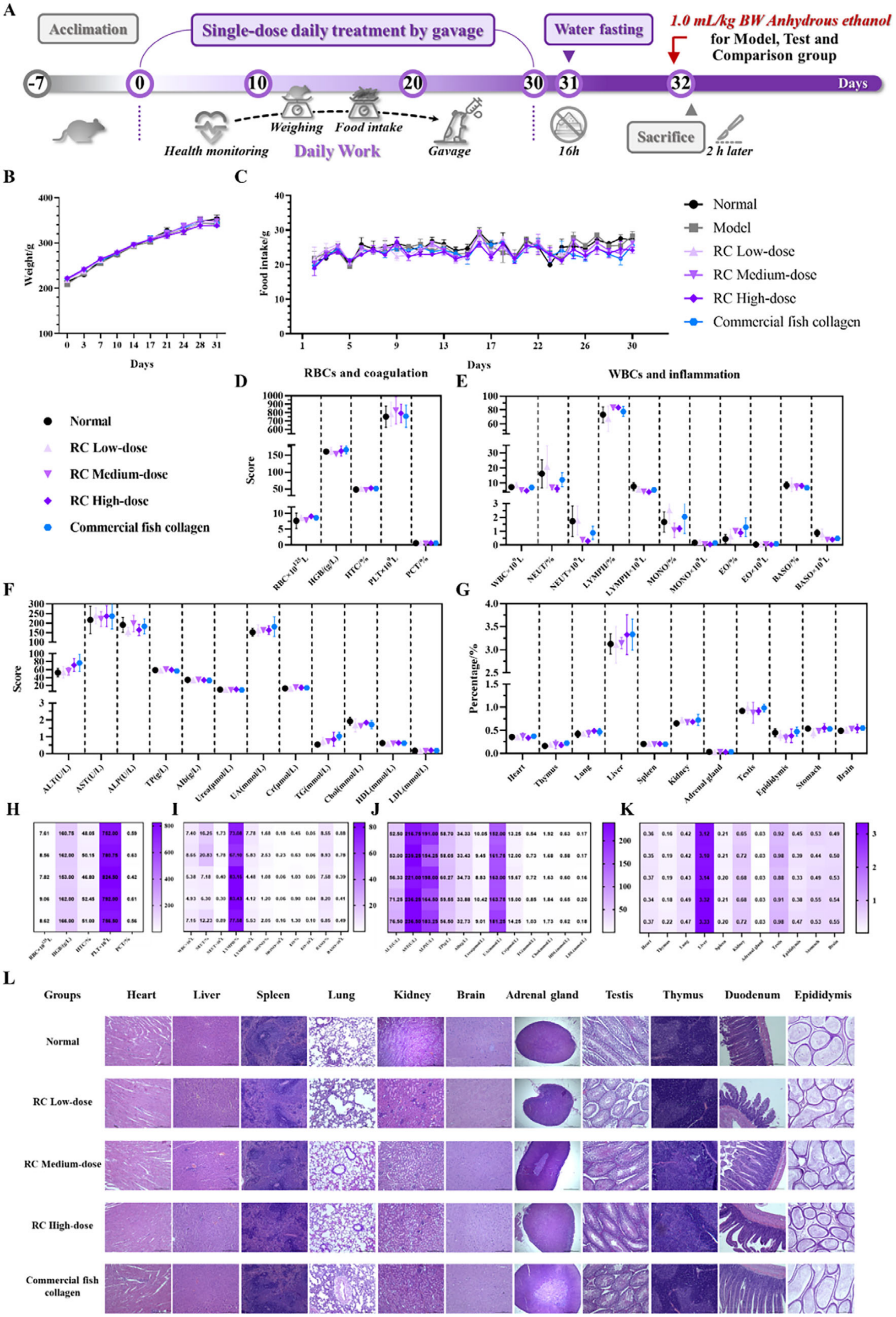
Safety evaluation of RC in rats. (A) Schematic overview of the experimental design. (B, C) Changes in body weight (B) and daily food intake (C) during the experimental period. (D) Red blood cell‐ and coagulation‐related hematological parameters. (E) White blood cell‐ and inflammation‐related hematological parameters. (F) Blood biochemical parameters. (G) Organ coefficients. (H–K) Heatmaps of the corresponding quantitative data shown in panels D–G, respectively. (L) Representative H&E‐stained histological images of major organs from the different treatment groups.

To assess the protective potential of RC against ethanol‐induced acute gastric mucosal injury (AGMI), after 7 days of adaptive feeding, healthy male Wistar rats were randomly assigned into 6 groups for different gavage treatments (*n* = 10): (i) Normal group (ii) Model group (iii) RC Low‐dose group (iv) RC Medium‐dose group (v) RC High‐dose group (vi) Commercial fish collagen group (same dose as RC High‐dose). The experimental period was 30 days to accomplish the preventive establishment of the gastric mucosal barrier in rats with AGMI (Figure 2A, Table S2).

The results showed that the trends of weight and daily food intake at different groups were in good agreement (Figure 2B,C), implying that the intake of RC at the test dose would not affect the normal feeding and digestion and absorption of rats. Further, there was no significant difference in blood routine indexes such as red blood cells, white blood cells and platelets between test and normal group (Figure 2D,E,H,I), suggesting that the RC did not cause inflammatory infection, blood diseases, coagulation dysfunction and so on pathological problems. Meanwhile, the blood biochemical indexes and organ coefficients of the test group were within the normal range (Figure 2F,J), as well as there were no significant pathological changes in the liver, kidney, brain, lung, digestive system (stomach, duodenum), hematopoietic system (spleen), immune system (thymus, spleen), reproductive system (testis, epididymis) in the tissue pathological slices (Figure 2G,K,L, Table S3), which proved that the RC had no obvious biological toxicity in the test dose and cycle.

**FIGURE 3:**
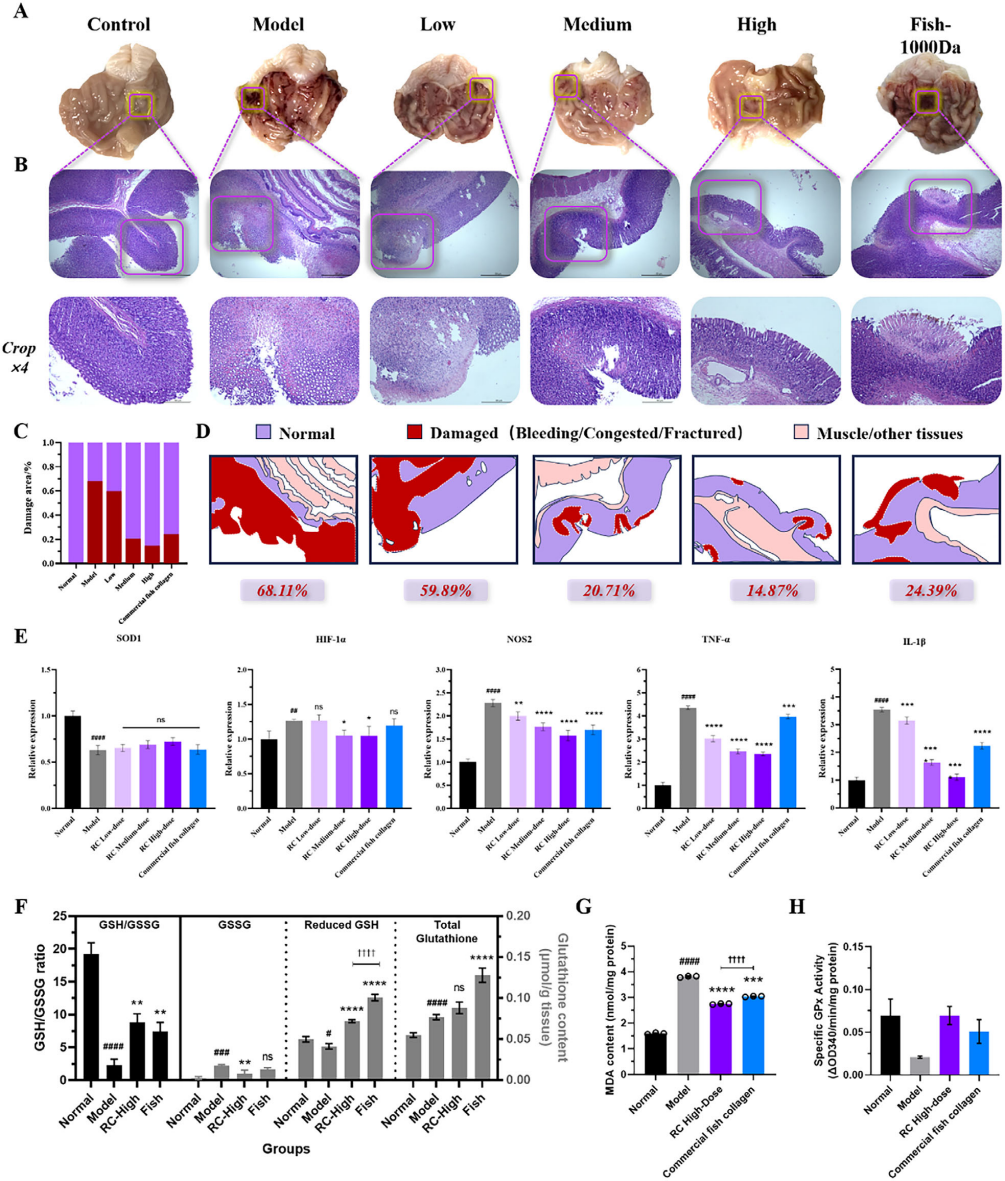
RC alleviates ethanol‐induced gastric mucosal injury and restores redox homeostasis in gastric tissue. (A) Representative photographs of the complete exposed gastric mucosal surfaces after the stomachs were opened along the greater curvature. (B) Low‐magnification overviews of H&E‐stained gastric tissue sections obtained from standardized lesion‐targeted sampling regions, together with higher‐magnification images of the corresponding boxed regions. (C) Percentage of microscopically damaged gastric mucosal area in different treatment groups. (D) Representative color‐coded segmentation used for the quantitative analysis in panel C, distinguishing normal mucosa, damaged regions, and muscle or other tissues. (E) Effect of RC treatment on the mRNA levels of SodD1, HifF‐1α, NOS2, TNF‐α, and IL‐1β in gastric tissue from ethanol‐induced AGMI rats. (F) Glutathione redox status in gastric tissue, including the GSH/GSSG ratio, GSSG, reduced GSH, and total glutathione. (G) MDA content in gastric tissue. (H) GPx activity in gastric tissue. For panels A‐E, data are presented as mean ± SEM (*n* = 4 per group), F‐G, data are presented as mean ± SEM (*n* = 4 per group), Statistical significance is indicated as ^#^ *P* < 0.05, ^##^ *P* < 0.01, ^###^ *P* < 0.001, and ^####^ *P* < 0.0001 compared with the Normal group; ^*^ *P* < 0.05, ^**^ *P* < 0.01, ^***^ *P* < 0.001, and ^****^ *P* < 0.0001 compared with the Model group; and ^†^ *P* < 0.05, ^††^ *P* < 0.01, ^†††^ *P* < 0.001, and ^††††^ *P* < 0.0001 for comparisons between the RC high‐dose and Fish groups. Scale bar: 100 µm.

After confirming the safety of RC, we further evaluated the preventive effect of each experimental group on ethanol‐induced AGMI with 1 mL/kg anhydrous ethanol. According to the macroscopic and microscopic scoring criteria presented in Tables S4 and S5, respectively, both the macroscopic and histopathological injury scores were significantly higher in the model group than in the normal group (*P* < 0.0001). Examination of the complete exposed gastric mucosal surface revealed extensive hemorrhagic lesions in the model group (Figure 3A). Consistently, H&E staining showed disruption, discontinuity, and defects of the gastric mucosal epithelium, and the damaged gastric mucosal tissue area reached 68.11% (Figure 3B–D). In Figure 3B, low‐magnification overviews of the tissue sections obtained using the standardized lesion‐targeted sampling procedure are presented together with higher‐magnification images of the corresponding boxed regions. These results confirmed the successful establishment of the ethanol‐induced AGMI model.

Correspondingly, the degree of gastric mucosal injury in the three RC‐treated groups showed a dose‐dependent response. The number of bleeding points, the length and width of bleeding bands, and the damaged gastric mucosal area decreased, with the increase of RC dose (Figure S5A–C), with significant differences observed between the model group and the RC medium‐ and high‐dose groups (Figure S5D–G). Especially in the high‐dose group, the gastric mucosal morphology differed markedly from that of the model group. The damaged gastric mucosal tissue area was substantially reduced, to approximately 20% of that observed in the model group, and the overall mucosal architecture was more continuous and regularly organized (Figure 3A–D). The low‐magnification histological overviews and the corresponding boxed higher‐magnification regions were consistent with these quantitative findings. These findings indicate that RC treatment provided dose‐dependent protection against ethanol‐induced AGMI. Comparatively speaking, the RC Low‐dose group showed only limited gastric mucosal protection, indicating that this dose was insufficient to provide substantial protection against ethanol‐induced injury. Moreover, it is worth noting that RC showed a better protective potential against ethanol‐induced AGMI than commercial fish collagen at the same dose, in both the macroscopic and histopathological evaluations. The macroscopic injury scores also differed significantly between the RC High‐dose and commercial fish collagen groups (P = 0.0359; purple significance bar in Figure S5E), further supporting the superior protective effect of RC under the tested conditions.

Therefore, we further quantified the mRNA expression of cytokines related to inflammatory injury in gastric mucosa of each experimental group to confirm the intervention degree of RC on AGMI. Figure 3E shows that in the model group, the expression of SOD1, indicates cellular antioxidant capacity, was significantly decreased, which could cause the excessive free radicals cannot be cleared by the antioxidant system and remain in the tissue, then a severe oxidative stress state is consequently present. Whereas the degree of up‐regulation of SOD1 in the RC group demonstrated a positive correlation with the intervening concentration, suggesting that the oxidative stress state in the tissues was alleviated. Meanwhile, HIF‐1α, as another indicator of cellular oxidative stress level, is highly expressed in the model group, suggesting that ethanol‐induced AGMI has caused severe oxidative stress. So that a significantly down‐regulate the expression of HIF‐1α at the statistical level only at a medium dose of RC undoubtedly proves its protective effect on AGMI. Furthermore, the expression of inflammatory response indicator factor NOS2, pro‐inflammatory cytokines TNF‐α and IL‐1β in model group were in striking contrast to the normal group, which was consistent with the gastric mucosal injury results observed by the naked eye and tissue sections (Figure 3A–C), suggesting acute and excessive ethanol intake is prone to cause gastric mucosa inflammation. What is noticed is that, as the RC dose gradient increased, the above factors’ expression gradually decreased, especially the high‐dose group exhibits higher efficacy, suggesting that this adverse inflammatory phenomenon of AGMI was effectively intervened. For the commercial fish collagen group, although the quantitative data of its antioxidant and inflammatory indicators also showed a certain resistance to AGMI, RC showed better protective ability under the same dose treatment, especially in reducing inflammation.

To further validate the restoration of gastric redox homeostasis at the biochemical level, glutathione redox status, lipid peroxidation, and antioxidant enzyme activity were directly assessed in gastric tissues from selected groups. Ethanol‐induced injury markedly disrupted the glutathione redox balance, as reflected by a pronounced decrease in the GSH/GSSG ratio together with increased GSSG levels in the Model group. RC High‐dose treatment partially restored the GSH/GSSG ratio, reduced GSSG accumulation, and increased reduced GSH levels (Figure 3F). In contrast, total glutathione displayed a more complex pattern and was not markedly altered by RC High‐dose treatment relative to the Model group, suggesting that the protective response was more closely associated with redistribution of the glutathione redox state than with a simple expansion of the total glutathione pool.

Consistent with the glutathione redox changes, MDA accumulation was markedly increased following ethanol‐induced injury and was reduced after RC High‐dose treatment, indicating attenuation of gastric tissue lipid peroxidation (Figure 3G). GPx activity also showed a recovery trend toward the Normal level in the RC High‐dose group, whereas commercial fish collagen showed a smaller recovery (Figure 3H). Representative calibration curves and kinetic profiles supporting these biochemical measurements are provided in Figure S6. Collectively, these direct tissue‐level biochemical measurements support the conclusion that RC alleviates ethanol‐induced oxidative stress by improving glutathione redox balance, reducing lipid peroxidation, and tending to restore endogenous antioxidant enzyme activity.

### 3.3 RC Remodels Gastric Tissue Metabolism and Activates an Arachidonic Acid‐Prostaglandin Pathway in Ethanol‐Induced AGMI

**FIGURE 4:**
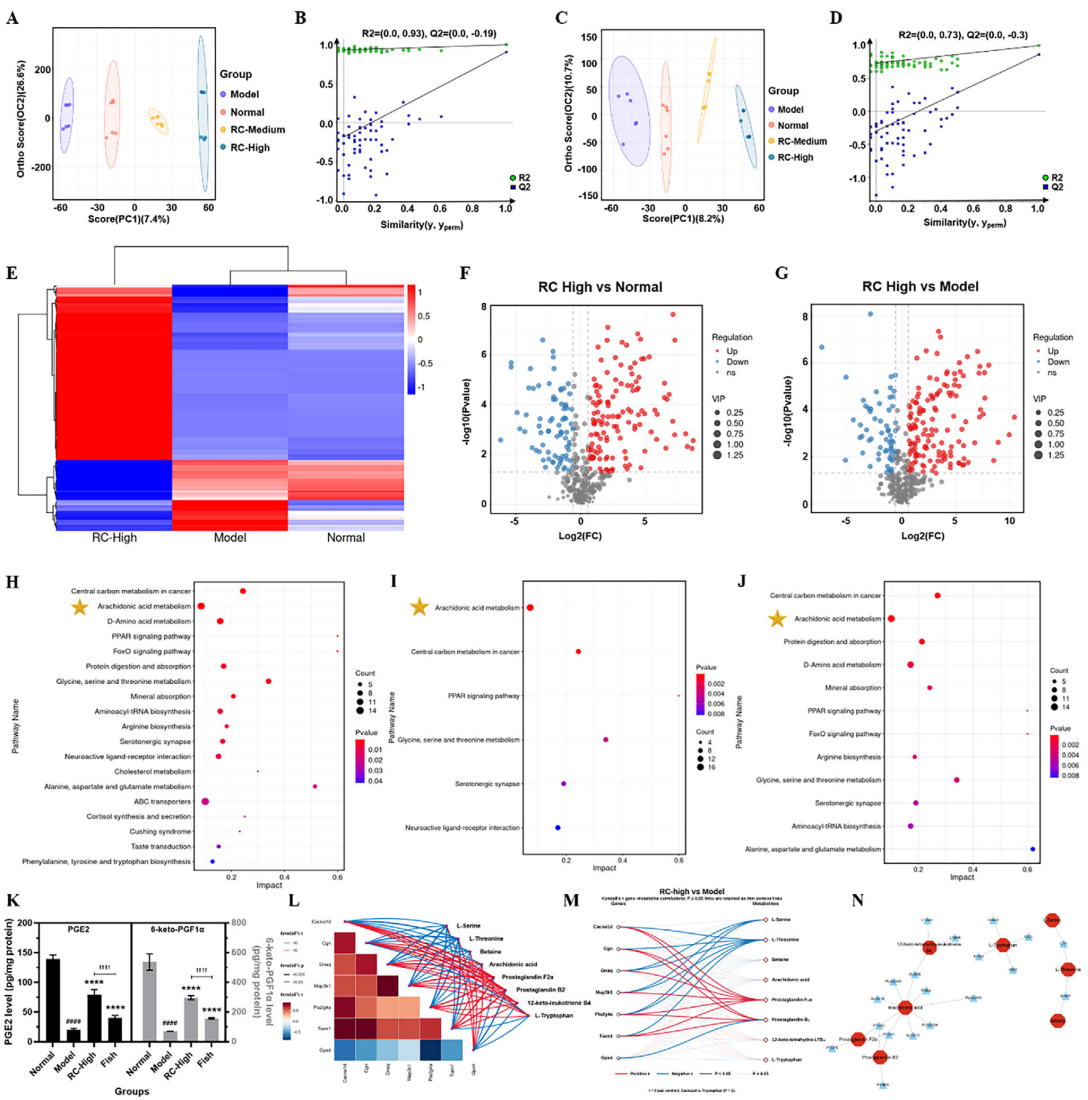
RC remodels the gastric tissue metabolome and reveals coordinated transcriptome‐metabolome associations. The score plots (A, C) and permutation (B, D) of OPLS‐DA among normal, model and RC‐Medium/High groups in the positive ion mode (A, B) and negative ion mode (C, D). (E) The heat map of differential metabolite analysis of RC‐high vs Model vs Normal group. The volcanic plot of differential metabolite analysis of (F) RC‐high vs Normal group and (G) RC‐high vs Model group. The bubble plot of differential metabolic pathways of (H) RC‐high vs Model vs Normal group, (I) RC‐high vs Normal group and (J) RC‐high vs Model group (*n* = 6 per group). (K) Targeted quantification of PGE2 and 6‐keto‐PGF1α in gastric tissue from the Normal, Model, RC High‐dose, and commercial fish collagen groups by competitive ELISA. PGE2 and 6‐keto‐PGF1α contents were normalized to total protein concentration and expressed as pg/mg protein (*n* = 4 per group). Statistical significance is indicated as ^####^ *P* < 0.0001 compared with the Normal group; ^****^ *P* < 0.0001 compared with the Model group; and ^††††^ *P* < 0.0001 for comparisons between the RC High‐dose and commercial fish collagen groups. (L) Integrated transcriptomic‐metabolomic correlation analysis for the RC‐high versus Model comparison. The WGCNA module‐metabolite heatmap shows the correlation coefficients and corresponding *P* values between gene co‐expression module eigengenes and selected differential metabolites. The Kendall's τ‐based butterfly plot shows the gene‐gene correlation matrix and gene‐metabolite associations; red and blue lines indicate positive and negative associations, respectively, and line width denotes the corresponding *P*‐value category. (M) Simplified bipartite view of the gene‐metabolite correlations shown in (L). Thick and thin links indicate *P* < 0.05 and *P* ≥ 0.05, respectively; the pair with τ = 0 was omitted. (N) Pathway association network linking selected metabolites and pathway‐related genes in key lipid‐ and amino acid‐metabolic pathways. Red octagons, blue triangles, and gray edges represent metabolites, genes, and pathway associations, respectively.

Non‐targeted metabolomics was performed on rat gastric tissues from the Normal, Model, RC‐medium, and RC‐high groups, followed by OPLS‐DA to assess group separation. RC treatment induced a clear dose‐dependent shift of the metabolic profile away from the ethanol‐induced Model group and toward the Normal group, indicating a marked remodeling of the gastric metabolome after 30 days of oral RC (Figure 4A–D). PCA and OPLS‐DA permutation tests confirmed the robustness and predictive performance of the models (Figure S9, Table S6).

Using VIP > 1.0 and *p* < 0.05 as cutoffs, we identified a set of significantly altered metabolites, which displayed distinct clustering patterns among the Normal, Model, and RC‐high groups (Figure 4E,G; Figure S10, Table S7). Pathway enrichment analysis based on KEGG revealed 19 significantly enriched pathways, spanning energy metabolism, membrane lipid remodeling, amino acid metabolism, and cell fate regulation (Figure 4H–J). Among these, arachidonic acid metabolism and glycine, serine and threonine metabolism emerged as key pathways that were consistently enriched when comparing RC‐high with both the Normal and Model groups.

Within arachidonic acid metabolism, RC‐high markedly increased a series of prostaglandin intermediates and end‐products, including PGF_2_α, 11β‐PGF_2_α, PGB_2_, and the leukotriene metabolite 12‐keto‐tetrahydro‐LTB_4_ (Figure 4E–G; Table S7). These prostaglandins and leukotrienes have been reported to promote vasodilation, enhance mucus and bicarbonate secretion, inhibit gastric acid secretion and platelet aggregation, and improve mucosal microcirculation, thereby protecting against gastric inflammatory injury and ulcers [10, 31, 32, 33, 34, 35, 36, 37]. The coordinated upregulation of these lipid mediators is therefore consistent with activation of a mucosal arachidonic acid‐prostaglandin pathway and restoration of prostaglandin‐dependent cytoprotection in the gastric mucosa.

To directly validate the downstream prostaglandin response indicated by the untargeted metabolomic analysis, PGE2 and 6‐keto‐PGF1α were quantified in gastric tissues from selected groups. Ethanol‐induced gastric mucosal injury markedly decreased both PGE2 and 6‐keto‐PGF1α levels compared with the Normal group. RC High‐dose treatment substantially restored both prostaglandin metabolites, whereas commercial fish collagen produced a more limited recovery. Moreover, the levels of both PGE2 and 6‐keto‐PGF1α were higher in the RC High‐dose group than in the commercial fish collagen group (Figure 4K). These targeted measurements directly corroborate the metabolomic evidence for restoration of the prostaglandin branch of arachidonic acid metabolism after RC treatment. The corresponding competitive ELISA calibration curves exhibited good fitting performance and are provided in Figure S7.

In parallel, RC‐high significantly modulated amino acid‐related pathways, including glycine, serine, and threonine metabolism, RC‐high also increased the levels of L‐tryptophan and betaine (Figure S10 and Table S7). L‐Tryptophan is a precursor of several bioactive metabolites, including melatonin, which has been implicated in upper gastrointestinal mucosal defense [15, 38]. Betaine is a physiological osmoprotectant and methyl donor with reported antioxidant, anti‐inflammatory, and intestinal barrier‐supporting properties [39, 40, 41]. These findings identify L‐tryptophan and betaine as RC‐associated metabolic alterations; however, their direct functional contribution to RC‐mediated gastric mucosal protection remains to be established.

Taken together, the untargeted metabolomic analysis and targeted quantification of PGE2 and 6‐keto‐PGF1α consistently support RC‐associated restoration of the arachidonic acid‐prostaglandin pathway in injured gastric tissue. In parallel, RC induced broader remodeling of amino acid‐related metabolism. These metabolic findings motivated the subsequent transcriptomic integration and mechanistic analyses, which focused on the *Pla2g4a*‐prostaglandin axis [35, 36, 37].

### 3.4 Transcriptomic Uncover RC‐Mediated Multi‐Mechanistic Coordination in Alleviating Ethanol‐Induced Gastric Mucosal Injury

**FIGURE 5:**
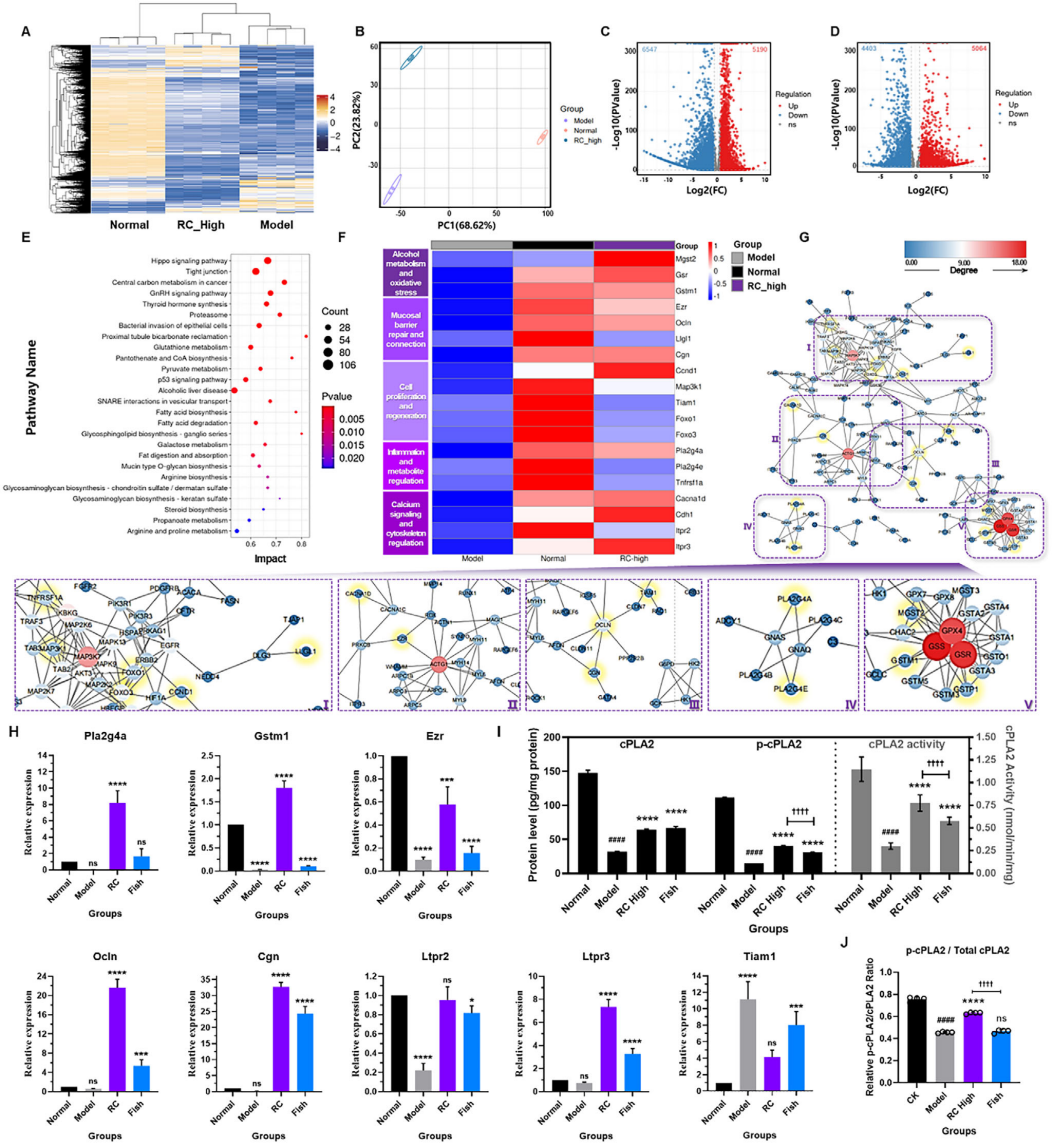
Transcriptomic landscape reveals coordinated RC‐mediated gastric mucosal repair and functional restoration of the *Pla2g4a*‐cPLA2 pathway. (A) The heatmap of difference gene of RC‐high vs Model vs Normal group. (B) PCA plot across RC‐high, Model and Normal group (PC1 vs PC2). Volcano plot of differentially expressed genes between (C) Model and Normal group, (D) RC‐high and Model group. (E) Bubble plot of enriched KEGG pathways. (F) Comparative heatmap of ethanol‐induced vs RC‐restored gene expression patterns across mucosal repair pathways. (G) Core PPI network of RC's gastroprotection. (H) qPCR validation of transcriptomic findings and comparative advantage of RC in modulating the *Pla2g4a*‐PG axis. (I) Total cPLA2 protein level, p‐cPLA2 level, and cPLA2 enzymatic activity in gastric tissues from the Normal, Model, RC High‐dose, and commercial fish collagen groups. Total cPLA2 and p‐cPLA2 levels were normalized to total protein and expressed as pg/mg protein, whereas cPLA2 activity was normalized to total protein and expressed as nmol/min/mg protein. (J) Ratio of p‐cPLA2 to total cPLA2, reflecting phosphorylation‐associated cPLA2 activation relative to total cPLA2 protein abundance. Data are presented as mean ± SEM (n = 4 per group for panels H‐J). Statistical significance is indicated as ^#^ *P* < 0.05, ^##^ *P* < 0.01, ^###^ *P* < 0.001, and ^####^ *P* < 0.0001 compared with the Normal group; ^*^ *P* < 0.05, ^**^ *P* < 0.01, ^***^ *P* < 0.001, and ^****^ *P* < 0.0001 compared with the Model group; and ^†^ *P* < 0.05, ^††^ *P* < 0.01, ^†††^ *P* < 0.001, and ^††††^ *P* < 0.0001 for comparisons between the RC High‐dose and commercial fish collagen groups.

Prompted by the metabolomic evidence for activation of an arachidonic acid‐prostaglandin pathway and remodeling of amino acid metabolism, we next sought to elucidate the upstream gene regulatory mechanisms engaged by RC. Transcriptomic analyses were therefore performed on gastric tissues from the Normal, Model, and RC‐high groups. Unsupervised hierarchical clustering of genes that passed expression and variability filters revealed highly consistent intra‐group expression patterns and clear inter‐group segregation, indicating robust transcriptional changes induced by ethanol and partially reversed by RC (Figure 5A). Principal component analysis (PCA) further showed distinct separation among the three groups, with RC‐high clustering closer to the Normal group than to the Model group along the principal components (Figure 5B), in line with the PCA trends observed in the metabolomic data.

Using *p* < 0.05 and fold change (FC) > 1.5 or FC < 0.67 as thresholds, we identified 11,737 differentially expressed genes (DEGs) in the Model vs Normal comparison, including 5190 upregulated and 6547 downregulated genes (Figure 5C). After 30 days of RC‐high intervention, the number of DEGs relative to the Model group decreased to 9467 (5,064 upregulated; 4403 downregulated) (Figure 5D), consistent with a partial normalization of ethanol‐induced transcriptional perturbations. KEGG enrichment analysis (*p* < 0.05, impact > 0.5) revealed 26 significantly enriched pathways, which could be broadly grouped into three functional categories: (i) immune and inflammatory regulation (e.g., B cell receptor and IL‐17 signaling pathways), (ii) metabolic adaptation (e.g., PPAR signaling, glycerolipid metabolism), and (iii) mucosal repair and neuroendocrine functions (e.g., tight junction, GnRH signaling) (Figure 5E).

To obtain a systems‐level view of RC‐mediated transcriptional regulation, we constructed a protein‐protein interaction (PPI) network based on DEGs and decomposed it into functionally coherent modules (Figure 5F,G). Three major modules were identified. First, a proliferation‐regeneration module, in which hub genes such as *Map3k7* and *Map3k1* connect with *CCND1* and the stress‐responsive transcription factors *FoxO1/3* to coordinate MAPK and FoxO signaling, pathways known to drive epithelial cell cycle progression and regenerative responses [42, 43, 44, 45, 46, 47, 48].

Second, a cytoskeleton‐barrier module, centered on *ACTG1* and including structural and junctional components such as *Ezr*, *Ocln*, *Cgn*, *Cdh1* and calcium signaling regulators including *Cacna1d* and *Itpr2/3*, consistent with their reported roles in maintaining epithelial polarity, tight junction integrity, and calcium‐dependent cytoskeletal dynamics [49, 50, 51, 52, 53, 54, 55, 56, 57, 58, 59, 60].

Third, an antioxidant metabolism module, in which *GPX4*, *GSS*, and *GSR* form a core complex that collaborates with glutathione pathway genes such as *Mgst2* and *GSTM1* to eliminate lipid peroxides and maintain glutathione homeostasis [61, 62, 63, 64, 65, 66]. Across these modules, RC‐high shifted the expression of key genes toward Normal‐like levels compared with the Model group, indicating coordinated restoration of proliferation/regeneration, barrier integrity, and redox balance (Figure 5F; Figure S11).

Within the inflammatory and lipid‐regulatory subnetworks, *Pla2g4a* and its isoform *Pla2g4e* emerged as notable nodes. RC‐high increased *Pla2g4a* expression relative to the Model group, and this upregulation was concordant with the elevated levels of prostaglandins (such as PGF_2_α, PGB_2_) detected in the metabolomic analysis. Cytosolic phospholipase A_2_ (cPLA_2_), encoded by *Pla2g4a*, is known to hydrolyze membrane phospholipids to release arachidonic acid, thereby supplying substrate for the downstream cyclooxygenase‐prostaglandin synthase cascade and driving prostaglandin biosynthesis [67, 68]. PPI analysis further indicated potential functional interactions between *Pla2g4a* and G‐protein‐related genes such as *GNAS* and *GNAQ* [69], which have been implicated in the regulation of arachidonic acid metabolism [70, 71]. Together with the metabolomic data, these observations identified the *Pla2g4a*‐prostaglandin axis as a major point of convergence between the RC‐responsive transcriptional and metabolic changes.

To determine whether the transcriptional changes identified above were concordant with the metabolic alterations observed in the same animals, we integrated the transcriptomic and metabolomic datasets. WGCNA revealed significant associations between gene co‐expression modules and the principal differential metabolites, including prostaglandins and amino acid‐related metabolites, while Kendall's correlation analysis further resolved specific gene‐metabolite relationships (Figure 4L–N). In the RC‐high versus Model comparison, *Pla2g4a* expression was positively correlated with PGF_2_α (Kendall's *τ* = 0.762, *P* = 0.0368) and PGB_2_ (*τ* = 0.905, *P* = 0.0046). These associations were directionally consistent with the concurrent upregulation of *Pla2g4a* and accumulation of prostaglandins identified independently by the transcriptomic and metabolomic analyses. Significant associations of *Cacna1d, Cgn, Gnaq, Map3k1*, *Tiam1*, and *Gpx4* with the selected metabolites further connected the differential metabolic profile with the calcium‐signaling, epithelial‐barrier, regenerative, and redox programs identified by transcriptome analysis. Pathway‐based network mapping placed these genes and metabolites within the arachidonic acid‐prostaglandin/leukotriene, tryptophan, and serine/threonine/betaine metabolic networks (Figure 4N). Collectively, the convergence observed at the pathway, co‐expression module, and individual gene‐metabolite levels provides cross‐omics corroboration for the reliability and biological coherence of the transcriptomic and metabolomic findings, with particularly strong support for RC‐induced activation of the *Pla2g4a*‐prostaglandin axis.

To validate the transcriptomic findings, we performed qPCR on representative genes from the above modules, including the inflammatory regulator *Pla2g4a*, cytoskeleton‐barrier components (*Ezr*, *Ocln*, *Cgn*), calcium signaling genes (*Itpr2*, *Itpr3*), and antioxidant/proliferation‐related genes (*Tiam1*, *GSTM1*). The expression trends observed by qPCR were consistent with the RNA‐seq results, and RC‐high exerted significantly stronger regulatory effects than commercial fish collagen at the same dose (Figure 5H). These qPCR results confirmed the reliability of the transcriptomic analysis and identified *Pla2g4a* as a transcriptionally responsive node within the RC‐regulated gastric mucosal repair network.

To further determine whether the transcriptional regulation of *Pla2g4a* was accompanied by protein‐level and functional activation, total cPLA2, p‐cPLA2, the p‐cPLA2/cPLA2 ratio, and cPLA2 enzymatic activity were directly assessed in gastric tissues. Ethanol‐induced gastric mucosal injury markedly reduced both total cPLA2 and p‐cPLA2 levels and suppressed cPLA2 enzymatic activity compared with the Normal group (Figure 5I). The p‐cPLA2/cPLA2 ratio was also decreased in the Model group, indicating that ethanol injury impaired not only cPLA2 abundance but also its relative phosphorylation‐associated activation (Figure 5J).

RC High‐dose treatment restored total cPLA2 protein abundance and p‐cPLA2 levels and significantly increased the p‐cPLA2/cPLA2 ratio compared with the Model group. Consistently, cPLA2 enzymatic activity was markedly recovered after RC High‐dose treatment. Commercial fish collagen also partially restored total cPLA2, p‐cPLA2, and enzymatic activity; however, it did not markedly improve the p‐cPLA2/cPLA2 ratio relative to the Model group. Moreover, RC High‐dose treatment produced greater increases in p‐cPLA2, the p‐cPLA2/cPLA2 ratio, and cPLA2 enzymatic activity than commercial fish collagen.

These findings extend the transcriptomic and qPCR observations from *Pla2g4a* mRNA regulation to cPLA2 protein abundance, activation‐associated phosphorylation, and direct enzymatic function. Together with the targeted PGE2 and 6‐keto‐PGF1α measurements, the results provide a continuous line of evidence supporting RC‐associated restoration of the *Pla2g4a*‐cPLA2‐prostaglandin pathway. The corresponding ELISA calibration curves are provided in Figure S8.

### 3.5 GPAEF Exhibits Prominent and G4‐Structure‐Dependent Modulation of the Pla2g4a Promoter G‐Quadruplex

Guided by the transcriptomic identification of *Pla2g4a* as a key node potentially linking RC treatment to activation of the arachidonic acid‐prostaglandin pathway, we next asked whether RC‐derived digestion products could directly modulate the G‐quadruplex in the *Pla2g4a* promoter region (*Pla2g4a*‐G4). In vitro simulated digestion experiments demonstrated that RC exhibited decent structural resistance in gastric juice. Although degradation commenced 2 min after exposure to gastric juice, distinct bands at the original protein position remained visible after 60 min (Figure S12), suggesting its potential to form a collagen barrier along the gastric wall for alcohol‐induced injury mitigation. Concurrently, based on our earlier finding that RC may exert gastroprotective effects via *Pla2g4a* regulation, we further performed peptidomic analysis and identified the GPAEF pentapeptide (Tables S8 and S9) as the most abundant and highest‐scoring fragment in 1‐h gastric fluid‐digested RC products [72]. To determine the gastric generation and temporal occurrence of GPAEF following oral RC administration, we performed a targeted LC‐MS/MS time‐course analysis covering 0–240 min. GPAEF was clearly confirmed in the gastric mucosal lavage collected at 30 min based on its retention time and three characteristic MRM transitions, with a mean concentration of 8.795 ng/mL. At 120 min, low‐intensity GPAEF‐related signals remained near the expected retention‐time window, although their responses were below the accepted calibration range and could not be reliably quantified. No confirmatory signal meeting all identification criteria was obtained from the remaining time points under the current analytical conditions. This temporal pattern was considered together with the simulated gastric digestion results, in which RC degradation began within 2 min while residual protein bands remained visible for up to 60 min, and GPAEF was subsequently identified among the 1‐h gastric digestion products. These findings indicate that RC undergoes progressive gastric digestion, producing a transient window during which GPAEF can accumulate to a quantifiable level before undergoing further degradation, dilution, or gastric emptying. Thus, the 0–240 min analysis provides direct in vivo evidence that oral RC gives rise to GPAEF in the gastric environment and that the released peptide reaches the mucus‐associated gastric mucosal surface (Figures S16 and S17; Tables S11 and S12). GPAEF was therefore prioritized as a representative candidate for subsequent in vitro biophysical analysis.

**FIGURE 6:**
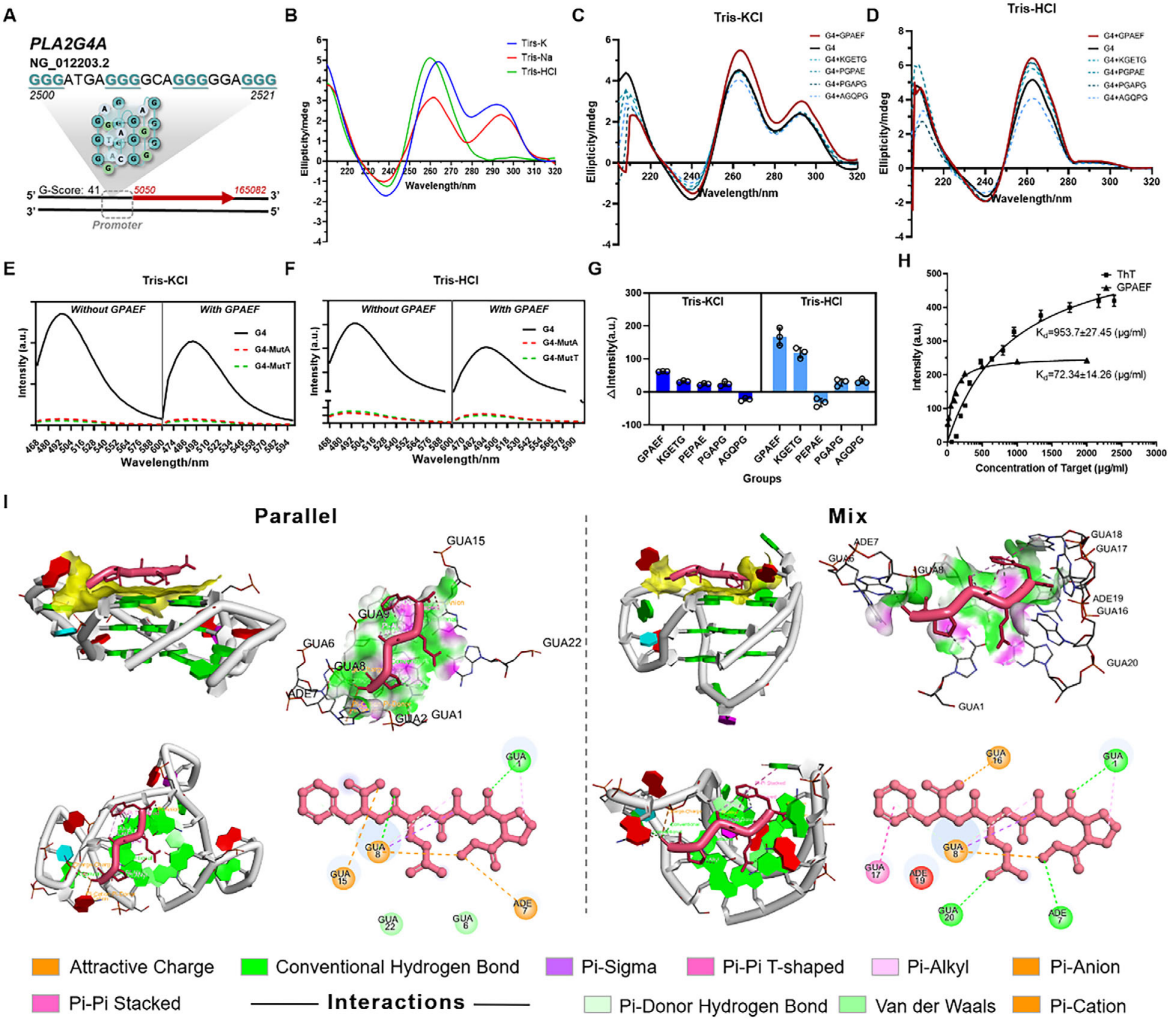
Comparative and mutant‐sequence analyses reveal prominent and G4‐structure‐dependent modulation of the *Pla2g4a* promoter G‐quadruplex by GPAEF. (A) Location, sequence, and schematic representation of the predicted G‐quadruplex‐forming region in the *Pla2g4a* promoter. (B) Ion‐dependent CD spectra of wild‐type *Pla2g4a*‐G4 in Tris‐KCl, Tris‐NaCl, and Tris‐HCl buffers. Comparative CD spectra of *Pla2g4a*‐G4 alone or after incubation with GPAEF, KGETG, PGPAE, PGAPG, or AGQPG under (C) Tris‐KCl and (D) Tris‐HCl conditions. ThT fluorescence emission spectra of wild‐type *Pla2g4a*‐G4, G4‐mutA, and G4‐mutT before and after GPAEF addition under (E) Tris‐KCl and (F) Tris‐HCl conditions. (G) Changes in ThT fluorescence intensity induced by the five pentapeptides under Tris‐KCl and Tris‐HCl conditions. ΔI was calculated as the fluorescence intensity before peptide addition minus that after peptide addition. (H) Determination of the apparent dissociation constants (K*_d_*) for the interactions of *Pla2g4a*‐G4 with GPAEF and ThT. GPAEF exhibited an apparent *K_d_* 13.18‐fold lower than that of ThT under the assay conditions. (I) Predicted intermolecular interactions between GPAEF and *Pla2g4a*‐G4 obtained by molecular docking.

To provide a matched comparison, four additional representative high‐abundance pentapeptides of the same chain length, KGETG, PGPAE, PGAPG, and AGQPG, were selected from the same peptidomic dataset. Using pentapeptides of identical length minimized potential differences arising from peptide chain length and molecular size. Accordingly, we investigated the G‐quadruplex within the *Pla2g4a‐*G4 (Figure 6A), a critical regulatory element in gene transcription containing specific secondary structures targetable by small molecules or peptides [73]. to determine whether GPAEF interacts with and modulates its G4 conformation in vitro.

*Pla2g4a*‐G4 was structurally characterized via G4Hunter prediction and circular dichroism spectroscopy. In Tris‐KCl, Tris‐NaCl, and Tris‐HCl buffers, canonical G4 signatures were observed: a 240 nm negative peak, 260/290 nm positive peaks (260 nm peak stronger), indicating a mixed‐type topology with prominent parallel features [74] (Figure 6B). To determine whether these spectral features depended on intact G‐tracts, two G4‐disrupting mutant sequences, G4‐mutA and G4‐mutT, were additionally examined. Both mutant sequences exhibited markedly altered or weakened characteristic G4‐associated CD features under Tris‐KCl and Tris‐HCl conditions, supporting impaired G4 folding following G‐tract mutation (Figure S14). The intrinsic CD spectra of the five free pentapeptides were first recorded to evaluate their individual spectral contributions. GPAEF concentration optimization showed that increasing peptide concentrations elevated the high‐tension voltage in the low‐wavelength region and reduced the valid scanning range. Therefore, 0.1 mg/mL was selected for subsequent peptide‐G4 interaction experiments. The intrinsic peptide spectra and concentration‐optimization results are provided in Figure S13.

Comparative CD spectroscopy was then performed to evaluate the effects of the five pentapeptides on *Pla2g4a‐G4* under identical conditions. Under Tris‐KCl conditions, GPAEF produced the most pronounced changes in the characteristic G4‐associated CD bands among the tested peptides (Figure 6C). A similar comparative pattern was observed in Tris‐HCl buffer, where the G4 structure was less strongly stabilized by monovalent cations (Figure 6D). KGETG, PGPAE, PGAPG, and AGQPG produced weaker or distinct spectral changes under the same conditions. These findings indicate that modulation of *Pla2g4a‐*G4 was not a general response shared equally by all abundant RC‐derived pentapeptides. The interaction mechanism was further validated by competitive fluorescence assays using the G4‐specific probe ThT [75]. Comparative ThT fluorescence analysis showed that GPAEF produced the largest change in ThT fluorescence intensity under both Tris‐KCl and Tris‐HCl conditions, whereas the other pentapeptides generated smaller, minimal, or directionally different responses (Figure 6G). Because ThT fluorescence depends on the formation and accessibility of its G4‐binding environment, these findings independently supported a particularly prominent effect of GPAEF on the folding state or ligand‐accessible surface of *Pla2g4a‐*G4.

The structural dependence of the GPAEF‐associated response was further evaluated using G4‐mutA and G4‐mutT. The wild‐type sequence displayed a pronounced change in its ThT emission spectrum following GPAEF addition. In contrast, the mutant sequences exhibited markedly altered basal ThT fluorescence and substantially attenuated responses to GPAEF under both Tris‐KCl and Tris‐HCl conditions (Figure 6E,F). Together with the mutant CD results, these observations support the interpretation that the GPAEF‐associated response depends on the integrity of the *Pla2g4a‐G4*‐forming sequence rather than representing a general peptide‐induced alteration in nucleic acid fluorescence. Fluorescence titration experiments further revealed that the apparent dissociation constant (K*_d_*) for GPAEF binding to *Pla2g4a*‐G4 was 13.18‐fold lower than that of ThT (Figure 6H), indicating a higher apparent binding affinity of GPAEF under the assay conditions. This result supports the ability of GPAEF to compete with ThT for the G4‐associated binding environment. Collectively, the peptide‐comparison and mutant‐sequence experiments indicate that GPAEF exhibits a relatively prominent and G4‐structure‐dependent interaction with *Pla2g4a‐G4* among the tested RC‐derived pentapeptides. These biophysical measurements support conformational modulation and stabilization of the G4 by GPAEF.

Then, parallel‐Q3 and Mixed‐Q5 (the mixed‐topology conformer with lowest clean energy (Figure S15), the lowest‐energy parallel and mixed‐topology conformers of *Pla2g4a*‐G4 were selected as receptors, with GPAEF as the ligand for molecular docking. In both topological conformers of *Pla2g4a*‐G4, a highly conserved binding motif was observed between GPAEF and three key nucleotides: G1 and G8 bases in the G‐tetrad core, and the adjacent A7 residue. Specifically, van der Waals interactions (= O) and Pi‐Alkyl stacking (pyrrolidine ring) between G1 and PRO enhanced hydrophobic stabilization, while Pi‐Sigma contacts at G8 provided additional binding anchorage. Crucially, conventional hydrogen bonds (G8‐GLU sidechain) and Pi‐Anion interactions (G8/A7 with GLY) collectively established a polar interaction network (Figure 6I). These cooperative noncovalent contacts may constrain local G4 conformational dynamics and favor peptide‐bound conformational states. The subsequent molecular dynamics simulations were therefore used to investigate the time‐dependent structural rearrangement and stability of the GPAEF‐G4 complex.

### 3.6 MD Simulation Elucidates the GPAEF‐Induced Conformational Remodeling Underlying Pla2g4a‐G4 Stabilization

Molecular dynamics (MD) simulations elucidated the dynamic stability and temporal evolution of the *Pla2g4a*‐G4/GPAEF complex, providing atomistic insights into the binding mechanism.

**FIGURE 7:**
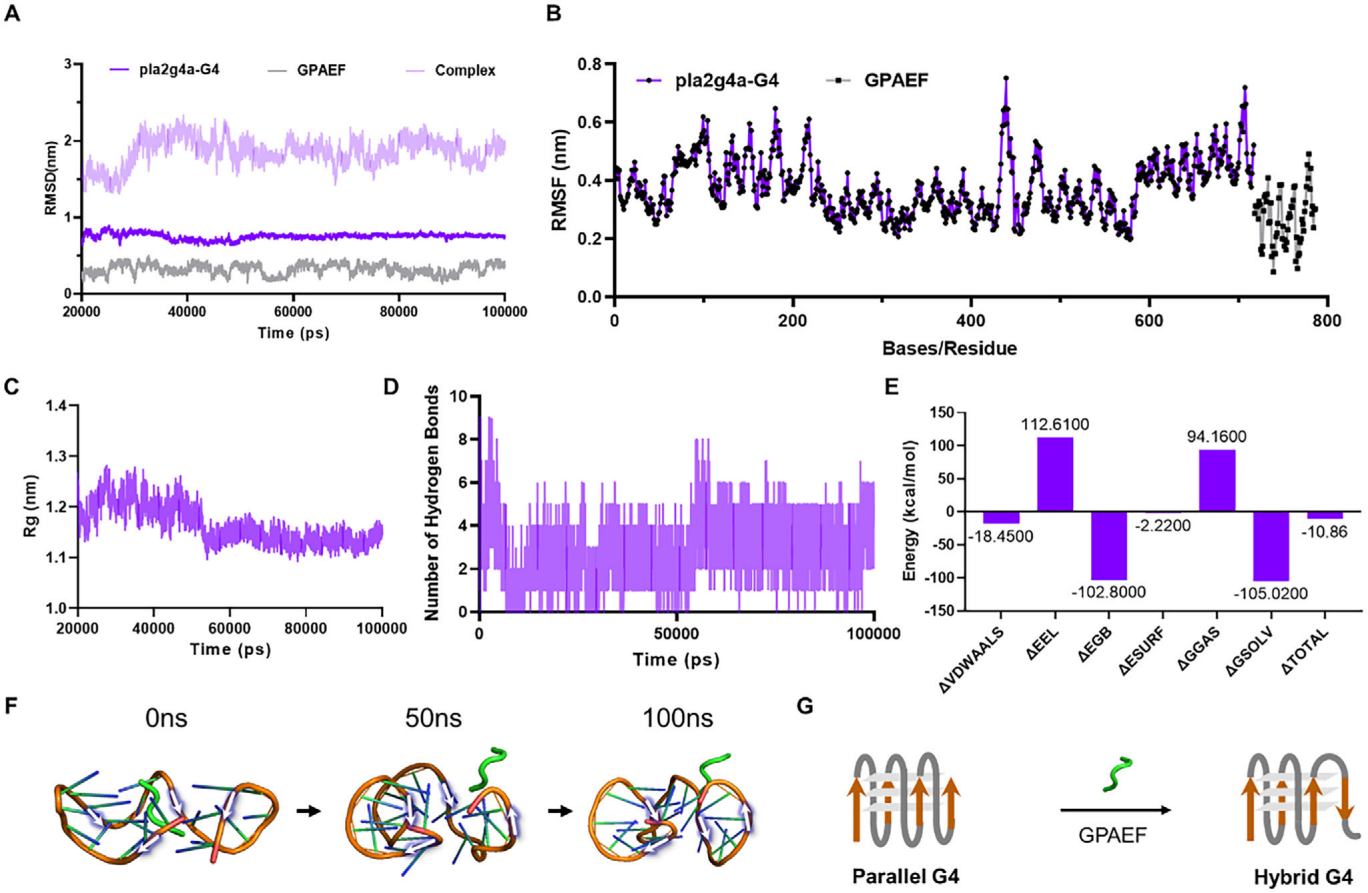
Molecular dynamics simulation reveals GPAEF‐mediated conformational remodeling and stabilization of the *Pla2g4a* promoter G‑quadruplex. (A) Time evolution of root‑mean‑square deviation (RMSD) for the initially modeled parallel G4‑GPAEF complex. (B) Residue‑level root‑mean‑square fluctuation (RMSF) of the complex. (C) Temporal change in the radius of gyration (Rg). (D) Evolution of the intermolecular hydrogen‑bond network between GPAEF and G4. (E) MM‐PBSA binding free energy decomposition for the initially modeled parallel G4‑GPAEF complex. (F) Superposition of representative structures at 0, 50, and 100 ns illustrating the conformational rearrangement of the initially modeled parallel G4 toward a mixed‐topology state. (G) Schematic diagram of the proposed GPAEF‑induced G4 conformational remodeling and stabilization.

Simulation of the initially modeled parallel G4‐GPAEF complex complex revealed a distinct conformational rearrangement. The root‐mean‐square deviation (RMSD) increased during the initial phase and converged after ∼31 ns, stabilizing at a plateau of ∼1.9 nm, indicative of a shift to a new conformational state (Figure 7A). Residue‐level root‐mean‐square fluctuation (RMSF) analysis then revealed the local dynamics underlying this transition. GPAEF demonstrated considerable structural rigidity (RMSF ∼0.2 nm), while the G4 sequence retained pronounced conformational flexibility (RMSF ∼0.3 nm). This distinct dynamic profile implies that GPAEF likely interacts with the structurally dynamic nucleic acid target through a local anchoring mechanism (Figure 7B). Concomitantly, global compaction was quantified via the radius of gyration. After initial fluctuations, the radius of gyration (Rg) underwent a sustained decrease, plateauing at ∼1.1 nm after ∼50 ns (Figure 7C), quantitatively capturing the transition to a more compact architecture. In parallel, the evolution of the intermolecular hydrogen‐bond network mirrored this structural transition. A dynamic phase (0‐50 ns) characterized by fluctuating hydrogen bonds (2‐8) was followed by a stable equilibrium phase with a consistent set of 4–6 specific hydrogen bonds (Figure 7D). Collectively, the stabilization of the compact Rg and the formation of a specific hydrogen‐bond network provided the molecular foundation for favoring a compact peptide‐bound mixed‐topology state.

Three representative structures at 0, 50, and 100ns characteristic points were selected to visualize the progressive conformational rearrangement of the initially modeled parallel G4 toward a hybrid/mixed‐like state (Figure 7F,G). The complete simulation trajectory over 0 to 100 ns further confirmed that this conformational rearrangement was sustained over the simulated timescale, rather than a transient fluctuation (Movie S1).

Finally, MM‐PBSA calculations unveiled the thermodynamic basis of this interaction, with a total binding free energy of –10.86 kcal/mol for the complex (Figure 7E). This energetically validates the high‐affinity binding between GPAEF and *Pla2g4a*‐G4.

In contrast, the mixed G4‐GPAEF complex maintained a highly stable architecture throughout the entire simulation without topological transitions (Figure S18A,B; Movie S2). Its Rg value, residue‐level fluctuations, and intermolecular hydrogen‐bond network remained stable over time (Figure S18B–D). Notably, the RMSF of the mixed‐G4 were consistently around 0.1 nm, markedly lower than those of the parallel G4, reflecting its inherent conformational rigidity. MM‐PBSA calculations further confirmed strong binding (−21.06 kcal/mol; Figure S18E).

Collectively, these results indicate that GPAEF remodels the mixed‐type Pla2g4a‐G4 conformational ensemble and favors a more compact hybrid/mixed‐like peptide‐bound state. This GPAEF‐mediated conformational remodeling biases the dynamic G4 ensemble toward a bound state with reduced conformational mobility and enhanced structural integrity. Stabilization of this regulatory G4 element may facilitate transcriptional initiation, for example, by promoting localized DNA pre‐melting at or near the transcription start site (Figure S19), and thereby contribute to the increased *Pla2g4a* expression observed in vivo. In turn, activation of the *Pla2g4a*‐PG pathway and enhanced production of protective prostaglandins provide a mechanistic connection between oral RC intake and reinforcement of the injury‐resistant gastric mucosal barrier.

In all, our MD results provide atomistic and time‐resolved support for the conclusion that GPAEF binds the *Pla2g4a*‐G4 with high affinity and promotes remodeling and stabilization of its mixed‐type conformational ensemble toward more compact peptide‐bound states, in agreement with the biophysical data. This dynamic conformational remodeling offers a mechanistic basis for the hypothesis that GPAEF modulates gene transcription through G4 stabilization (Figure 7G).

## 4 Discussion

This study is based on the global public health challenge of ethanol‐induced AGMI, systematically investigating the dose‐response relationship and mechanisms of RC in this pathological context, and thereby supporting its potential as a human‐derived biomaterial with novel applications. Our study demonstrated that RC intervention elicits a dose‐dependent reduction in oxidative stress accompanied by recovery of mitochondrial membrane potential in GES‐1 cells, suggesting it alleviates ethanol‐induced damage by preserving mitochondrial function. The optimal dosage reduces mucosal lesion area by 78% versus model controls, SOD activity was increased nearly by 40%, and decreases pro‐inflammatory cytokines (e.g., IL‐1) by 55%, confirming its multi‐target efficacy in antioxidation, mucosal protection, and inflammation regulation. Crucially, no traditional drug‐associated side effects (e.g., hematological/immunological parameter alterations or hepatorenal toxicity) were observed, underscoring its biosafety. Notably, RC maintains relatively stable structural integrity for 60 min in vitro simulated gastric acid digestion models, while exhibiting 1.5‐fold greater anti‐injury effect than fish collagen peptides in vivo. These findings not only fill a research gap regarding RC in gastrointestinal mucosal protection, but also reveal the unique advantages of macromolecular collagens in long‐term tissue support and adaptation to complex microenvironments.

Furthermore, gastric tissue metabolomic profiling revealed broad RC‐associated metabolic remodeling, prominently involving activation of the arachidonic acid‐prostaglandin biosynthesis pathway, together with additional changes in amino acid‐related metabolites, including L‐tryptophan and betaine. Because the functional roles of the latter metabolites were not directly tested, they are interpreted as exploratory RC‐associated metabolic alterations rather than established mediators of gastric mucosal protection. Then, PPI network dissection uncovered a multi‐modular cooperation: Map3k7‐mediated proliferation/redox coordination, ACTG1‐driven cytoskeleton‐barrier assembly, and GPX4‐GSS‐GSR antioxidant core, synergistically enabling mucosal regeneration. These transcriptomic findings were further supported by direct gastric tissue biochemical measurements, which showed partial restoration of the GSH/GSSG ratio, reduced GSSG and MDA accumulation, and a recovery trend in GPx activity after RC High‐dose treatment.

Importantly, the integrated transcriptomic‐metabolomic analysis further reinforced this interpretation by showing that the metabolic pathways identified by untargeted profiling were accompanied by coordinated transcriptional changes. In particular, the positive associations of Pla2g4a with PGF_2_α and PGB_2_, together with their concordant increases following RC‐high treatment, provide cross‐omics support for activation of the arachidonic acid‐prostaglandin cascade. The additional module‐ and gene‐level associations involving calcium signaling, epithelial‐barrier integrity, redox regulation, and amino acid‐related metabolism further indicate that RC‐induced gastric mucosal protection is accompanied by coordinated remodeling across the transcriptional and metabolic layers.

Crucially, the *Pla2g4a*‐PG axis was identified as a regulatory node linking phospholipid hydrolysis to arachidonic acid metabolic activation. Direct gastric tissue measurements further showed that ethanol‐induced injury reduced total cPLA2 protein abundance, p‐cPLA2 levels, the p‐cPLA2/cPLA2 ratio, and cPLA2 enzymatic activity. RC High‐dose treatment restored total cPLA2 and p‐cPLA2 levels, increased the p‐cPLA2/cPLA2 ratio, and recovered cPLA2 catalytic activity, thereby extending the transcriptomic regulation of Pla2g4a to protein abundance, phosphorylation‐associated activation, and enzymatic function. Although commercial fish collagen partially restored total cPLA2, p‐cPLA2, and cPLA2 activity, RC High‐dose treatment produced a greater recovery of p‐cPLA2 levels, the p‐cPLA2/cPLA2 ratio, and enzymatic activity. Direct quantification of PGE2 and 6‐keto‐PGF1α further supported this pathway‐level interpretation, showing that both representative downstream prostaglandin metabolites were markedly depleted following ethanol‐induced injury and substantially restored after RC High‐dose treatment. The stronger recovery observed with RC High‐dose than with commercial fish collagen was consistent with the greater gastric mucosal protection provided by RC under the tested conditions. Collectively, the transcriptomic, protein‐level, phosphorylation, enzymatic, and targeted metabolite results provide a continuous line of evidence supporting RC‐associated restoration of the *Pla2g4a*‐cPLA2‐prostaglandin pathway. Nevertheless, these findings support the involvement of this pathway as one contributing component of RC‐mediated gastroprotection and do not establish it as the sole mediator of the overall protective effect.

At the upstream regulatory level, GPAEF, a gastric‐digested RC pentapeptide, was further investigated as one potential contributor to this pathway through its interaction with the *Pla2g4a* promoter G4.

An essential prerequisite for this proposed upstream mechanism is that GPAEF is generated from orally administered RC and becomes available at the gastric mucosal interface. Consistent with the progressive RC digestion observed in vitro, targeted LC‐MS/MS analysis over 0–240 min confirmed GPAEF in mucus‐associated gastric lavage at 30 min, with a mean concentration of 8.795 ng/mL, while low‐intensity GPAEF‐related signals remained detectable but unquantifiable at 120 min. The non‐continuous recovery of GPAEF likely reflects the dynamic balance among its generation from RC, further proteolytic degradation, heterogeneous distribution and adsorption within gastric contents and mucus, dilution during lavage, gastric emptying, matrix interference, and limited analytical sensitivity at trace concentrations. Therefore, failure to confirm GPAEF at an individual time point does not necessarily indicate its complete biological absence. Together with the simulated digestion kinetics and peptidomic identification of GPAEF in gastric digestion products, the confirmed 30‐min signal provides direct in vivo evidence that oral RC gives rise to GPAEF in the gastric environment and that the released peptide reaches the mucus‐associated gastric mucosal surface, thereby supporting the subsequent investigation of the GPAEF‐*Pla2g4a*‐G4 regulatory mechanism.

Among the five representatives high‐abundance RC‐derived pentapeptides examined under matched experimental conditions, GPAEF induced the most pronounced changes in the characteristic CD signals and ThT fluorescence response of *Pla2g4a*‐G4. The weaker or distinct responses produced by KGETG, PGPAE, PGAPG, and AGQPG indicate that the observed modulation was not a nonspecific property shared equally by all abundant pentapeptides released from RC. Consistently, fluorescence titration showed that GPAEF exhibited an apparent dissociation constant (K*_d_*) 13.18‐fold lower than that of ThT, supporting its comparatively strong interaction with the *Pla2g4a*‐G4 binding environment.

The G4‐disrupting mutant sequences provided an additional structural control. Mutations within the G‐tracts markedly altered the characteristic CD features of *Pla2g4a*‐G4 and substantially attenuated the ThT response to GPAEF. These findings support a G4‐structure‐dependent interaction rather than a general peptide effect on nucleic acid fluorescence. Nevertheless, the present biophysical analyses evaluate peptide‐G4 interactions in vitro and do not establish that GPAEF is the only biologically active RC‐derived peptide or that the *Pla2g4a*‐G4 pathway is indispensable for the overall gastroprotective effect of RC.

The combined CD, ThT, binding‐affinity, docking, and molecular dynamics results are most consistent with GPAEF‐induced conformational modulation and redistribution of the *Pla2g4a*‐G4 ensemble. Accordingly, the structural response is interpreted as stabilization‐associated conformational remodeling and redistribution of the G4 ensemble rather than complete strand unwinding. GPAEF is instead interpreted as a representative RC‐derived pentapeptide with prominent *Pla2g4a*‐G4‐modulating activity, and this interaction is positioned as one experimentally supported upstream component of the broader *Pla2g4a*‐cPLA2‐prostaglandin pathway.

To our knowledge, these findings provide an example of a biomaterial‐derived digestion peptide exhibiting sequence‐ and structure‐dependent interaction with a promoter G‐quadruplex, thereby offering a testable molecular framework for investigating peptide‐mediated regulation of gastric mucosal gene expression. Notably, our experimental data reveal that the *Pla2g4a*‐G4 in solution exhibits spectral features consistent with a mixed‐type topology with prominent parallel characteristics, suggesting it populates a dynamic conformational equilibrium rather than a single, discrete state. MD simulations further suggest that GPAEF preferentially stabilizes more compact hybrid/mixed‐like peptide‐bound conformations within this ensemble. These findings are consistent with GPAEF‐induced redistribution of the G4 conformational ensemble toward more compact peptide‐bound states, while highlighting the structural plasticity of the G4 as a potential basis for its regulatory behavior.

It is important to acknowledge the discrepancies observed between the static molecular docking and MD simulation results (Figure 6I vs. Figure S20), which may partly stem from differences in docking protocols and scoring algorithms. Nevertheless, despite differences among the initial docking poses, the simulations consistently showed persistent peptide‐G4 contacts and convergence toward compact peptide‐bound conformational states. The time‐resolved MD results therefore complement the static docking models by illustrating the dynamic rearrangement and stabilization of the GPAEF‐G4 complex, while remaining a computationally supported mechanistic model that requires further validation in cellular chromatin contexts.

Although the revised data provide multi‐level evidence supporting activation of the *Pla2g4a*‐prostaglandin axis, direct loss‐of‐function evidence is not available in the present study. Therefore, the functional necessity of *Pla2g4a* for RC/GPAEF‐mediated gastric mucosal protection remains to be established.

Separately, the present study was designed as a 30‐day preventive oral intervention before ethanol challenge, rather than as a therapeutic drug‐comparison study. Accordingly, commercial fish collagen was included as a category‐matched material comparator, whereas a positive pharmacological control was not incorporated into the original experimental design. Therefore, the relative efficacy of RC compared with established gastroprotective drugs remains to be determined through a dedicated contemporaneous head‐to‐head study.

However, building upon current findings, the clinical translation of RC necessitates resolution of three pivotal challenges: (1) Although the present lavage‐based time‐course analysis supports the transient gastric occurrence of GPAEF, more comprehensive studies are required to define the full degradation kinetics of RC and the mucosal uptake, intracellular distribution, and clearance of GPAEF. (2) Interactions with gastrointestinal metabolic networks such as acetaldehyde‐induced conformational changes, microbiota‐derived metabolite regulation needs multi‐omics integration. (3) the intervention potential and molecular marker spectrum of RC in chronic lesions like ulcerative lesions have not been established, which limits the precise expansion of its clinical indications.

Therefore, it can be seen that the future directions include: (1) Smart Delivery Systems: Develop like pH‐sensitive hydrogels for targeted RC release with enhanced enzymatic stability, potentially combined with prostaglandin analogs/phyto‐antioxidants for multi‐target therapy. (3) Function‐Optimized Design: Engineer RC via synthetic biology (functional domain modification such as aptamer, antibody conjugation et al.) to improve gastric epithelial/mucosal cell targeting, and potentially enrich peptide motifs with high affinity for defined promoter G4s to expand the scope of transcriptional regulation. (4) Multi‐Omics Mechanism: Integrate spatial metabolomics, single‐cell transcriptomics, and metagenomics to decode RC's spatiotemporal effects across epithelial/fibroblast/immune cell subsets and its metabolic homeostasis regulation. (5) Advanced Models: Establish 3D gastric organoids and chronic injury models to evaluate long‐term protective efficacy.

In summary, this study demonstrates that genetically engineered RC is a promising preventive oral biomaterial with favorable biosafety and gastroprotective efficacy against ethanol‐induced AGMI. With its well‐defined molecular conformation and non‐immunogenic properties, RC provides a mechanistically distinct preventive approach involving coordinated regulation of redox homeostasis, barrier integrity, and mucosal regeneration, while showing advantages over the category‐matched commercial fish collagen comparator evaluated in this study. Integrated transcriptomic‐metabolomic analysis, together with cPLA2 protein/activity measurements and targeted prostaglandin quantification, provided convergent evidence for RC‐associated activation of the *Pla2g4a*‐cPLA2‐prostaglandin pathway, while the sequence‐ and G4‐structure‐dependent interaction between GPAEF and the *Pla2g4a* promoter G4 offers a plausible upstream regulatory basis for this response. Interestingly, RC exhibits unique temperature‐responsive behavior: it self‐assembles into a three‐dimensional hydrogel network at 4°C while reverting to liquid formulation properties at 25°C (Figure S21). Its temperature‐sensitive phase transition property not only provides technical feasibility for cold chain systems (including low‐temperature production, packaging, and transportation) to extend shelf life, but also maintains solution homogeneity during oral delivery for precise mucosal coverage. These advantages endow RC with superior application prospects in gastric mucosal injury protection and repair.

These findings support the further development of RC as a promising food‐grade oral biomaterial candidate for preventive gastric mucosal protection.

## Funding

This work was supported by the Internal Horizontal Service Contract of China Agricultural University entitled Study on Repair Test of Collagen for Gastric Mucosal Injury (Grant No. [202305523140776]), sponsored by Beijing Nova Program (Grant No. [20230484463]).

## Conflicts of Interest

The authors declare no conflicts of interest.

## Supporting information




**Supporting File 1**: advs77964‐sup‐0001‐SuppMat.docx.

**Supporting File 2**: advs77964‐sup‐0002‐SuppMat.zip.

**Supporting File 3**: advs77964‐sup‐0003‐SuppMat.zip.

## Data Availability

The data that supports the findings of this study are available in the supplementary material of this article.
