# Supplementary material for: Oral Recombinant Human Collagen III Protects Gastric Mucosa via G‐Quadruplex‐Mediated Activation of the Pla2g4a‐Prostaglandin Axis
Source: Adv Sci (Weinh). 2026 Sep 27:e77964. Online ahead of print. doi: 10.1002/advs.77964 (PMC13616389; doi:10.1002/advs.77964)
Supplement: Supplementary file 1 [file ADVS-9999-e77964-s001.docx]

**Supplementary Materials**

**Oral Recombinant Human Collagen III Protects Gastric Mucosa via G-Quadruplex-Mediated Activation of the Pla2g4a-Prostaglandin Axis**

Xinyue Lan^b,1^, Qiu Dai^a,1^, Yanhui Wang^b,1^, Jiaxuan Gu^b^, Chengyun Wang^b^, Weicheng Xu^b^, Xinru Gao^b^, Jiageng Cheng^a,b^, Wentao Geng^b^, Xiaowen Wu^c^, Haihang Li^c^, Xiaoyun He^a^, Shengwei Fu^c,^*, Longjiao Zhu^b,^*, Wentao Xu^b,^*

^a^ Key Laboratory of Precision Nutrition and Food Quality, Beijing Laboratory for Food Quality and Safety, College of Food Science and Nutritional Engineering, China Agricultural University, Beijing 100083, PR China;

^b^ Food Laboratory of Zhongyuan, Key Laboratory of Precision Nutrition and Food Quality, Department of Nutrition and Health, China Agricultural University, Beijing 100193, PR China;

^c^ Jiangsu Trautec Medical Technology Co., Ltd. Jiangsu 213200, PR China;

^1^ These authors contributed equally to this work.

* Corresponding authors:

Prof. Wentao Xu ([xuwentao@cau.edu.cn](mailto:xuwentao@cau.edu.cn))

Prof. Longjiao Zhu (zhulongjiao@126.com)

Ph.D Shengwei Fu (fushengwei@trautec.com.cn)

| Experiment Type | Dosage | Converted to μg/mL | Experiment Type |
| --- | --- | --- | --- |
| Cell-*GES1* | 450 μg/mL | 450 μg/mL | 1× |
| Animal-*Rat* | 0.9 g/kg | 900 μg/mL | 2× |
|  | 4.5 g/kg | 4,500 μg/mL | 10× |
|  | 22.5 g/kg | 22,500 μg/mL | 50× |

**Table S1. Dose conversion (In vitro-in vivo)**

| Group | Control group | | Test group | | | Comparison group |
| --- | --- | --- | --- | --- | --- | --- |
|  | Normal  (Negative) | Model  (Positive) | Low | Medium | High | Commercial fish collagen |
| Subject | ddH_2_O for 30days and 1.0 mL/BW Anhydrous ethanol only for Model in 2 hours before execution. | | Recombinant collagen | | | Fish collagen 1000Da |
| Dose（g/kg·BW） | 20 mL/kg | | 0.18 | 0.9 | 4.5 | 4.5 |
| Treatment | Gavage treatment once daily for 30 days | | | | | |

**Table S2. Group settings and experimental treatments**

**Table S3. Organs wet weight of rats in each group**

| **Organ** | **Normal** | | | **Model** | | | | **RC Low-dose** | | | | **RC Medium-dose** | | | | **RC High-dose** | | | **Commercial fish collagen** | | | | |
| --- | --- | --- | --- | --- | --- | --- | --- | --- | --- | --- | --- | --- | --- | --- | --- | --- | --- | --- | --- | --- | --- | --- | --- |
|  | **‾X** | SD | N | | ‾**X** | SD | N | | ‾**X** | SD | N | | ‾**X** | SD | N | | **‾X** | SD | | N | ‾**X** | SD | N |
| **Heart** | 1.26 | 0.05 | 10 | | 1.25 | 0.11 | 10 | | 1.21 | 0.10 | 10 | | 1.28 | 0.27 | 10 | | 1.14 | 0.06 | | 10 | 1.29 | 0.06 | 10 |
| **Thymus** | 0.57 | 0.05 | 10 | | 0.67 | 0.05 | 10 | | 0.66 | 0.07 | 10 | | 0.68 | 0.33 | 10 | | 0.60 | 0.19 | | 10 | 0.77 | 0.14 | 10 |
| **Lung** | 1.48 | 0.26 | 10 | | 1.87 | 0.68 | 10 | | 1.46 | 0.08 | 10 | | 1.50 | 0.27 | 10 | | 1.65 | 0.09 | | 10 | 1.60 | 0.27 | 10 |
| **Liver** | 11.03 | 0.78 | 10 | | 11.14 | 1.78 | 10 | | 10.69 | 1.36 | 10 | | 10.93 | 0.43 | 10 | | 11.24 | 1.47 | | 10 | 11.46 | 1.16 | 10 |
| **Spleen** | 0.73 | 0.13 | 10 | | 0.71 | 0.14 | 10 | | 0.73 | 0.11 | 10 | | 0.69 | 0.09 | 10 | | 0.71 | 0.15 | | 10 | 0.69 | 0.08 | 10 |
| **Kidney** | 2.29 | 0.07 | 10 | | 2.42 | 0.08 | 10 | | 2.47 | 0.21 | 10 | | 2.36 | 0.20 | 10 | | 2.31 | 0.14 | | 10 | 2.48 | 0.42 | 10 |
| **Adrenal gland** | 0.12 | 0.01 | 10 | | 0.09 | 0.02 | 10 | | 0.09 | 0.04 | 10 | | 0.10 | 0.02 | 10 | | 0.10 | 0.06 | | 10 | 0.12 | 0.05 | 10 |
| **Testis** | 3.25 | 0.19 | 10 | | 3.14 | 0.27 | 10 | | 3.39 | 0.42 | 10 | | 3.07 | 0.79 | 10 | | 3.09 | 0.24 | | 10 | 3.38 | 0.28 | 10 |
| **Epididymis** | 1.58 | 0.32 | 10 | | 1.22 | 0.42 | 10 | | 1.34 | 0.37 | 10 | | 1.15 | 0.32 | 10 | | 1.29 | 0.49 | | 10 | 1.63 | 0.35 | 10 |
| **Stomach** | 1.89 | 0.14 | 10 | | 1.89 | 0.27 | 10 | | 1.50 | 0.35 | 10 | | 1.69 | 0.16 | 10 | | 1.86 | 0.31 | | 10 | 1.83 | 0.17 | 10 |
| **Brain** | 1.74 | 0.16 | 10 | | 1.76 | 0.11 | 10 | | 1.73 | 0.24 | 10 | | 1.84 | 0.14 | 10 | | 1.84 | 0.30 | | 10 | 1.88 | 0.08 | 10 |

**Table S4.** **The visual observation scoring criteria of AGMI**

| **Degree of injury** | **1 point** | **2 points** | **3 points** | **4 points** |
| --- | --- | --- | --- | --- |
| **[*A*]** Numbers of Bleeding point | 1 | 2 | 3 | ≥4 |
| **[*B*]** Length of bleeding band | 1-5mm | 6-10mm | 10-15mm | ＞15mm |
| **[*C*]** Width of Bleeding band | 1-2mm | ＞2mm | - | - |
| **Total score=*A*+*B*+*C*×2** | | | | |

**Table S5.** **The** **microscopic scoring criteria of AGMI**

| **Degree of injury** | **1 point** | **2 points** | **3 points** | **4 points** | **5 points** |
| --- | --- | --- | --- | --- | --- |
| **[*A*]** Hyperaemia | 1/5 | 1/5-2/5 | 2/5-3/5 | 3/5-4/5 | Full epithelial layer |
| **[*B*]** Hemorrhage | 1/5-2/5 | 1/5-2/5 | 2/5-3/5 | 3/5-4/5 |  |
| **[*C*]** Epithelial cell necrosis | 1/5-2/5 | 1/5-2/5 | 2/5-3/5 | 3/5-4/5 |  |
| **Total score=*A*+*B*×2+*C*×3** | | | | |  |

**Table S6. The model parameter of OPLS-DA**

| **Mode** | **Group** | **A** | **R^2^X(cum)** | **R^2^Y(cum)** | **Q^2^(cum)** |
| --- | --- | --- | --- | --- | --- |
| Positive | Model vs Normal | 6 | 0.97 | 1 | -0.03 |
|  | Model vs RC-Medium | 6 | 0.84 | 1 | -0.20 |
|  | Model vs RC-High | 6 | 0.87 | 1 | -0.21 |
|  | RC-Medium vs Normal | 6 | 0.86 | 1 | -0.14 |
|  | RC-High vs Normal | 6 | 0.84 | 1 | -0.12 |
|  | RC-Medium vs RC-High | 6 | 0.85 | 1 | -0.15 |
| Negative | Model vs Normal | 6 | 0.87 | 1 | -0.23 |
|  | Model vs RC-Medium | 6 | 0.82 | 1 | -0.20 |
|  | Model vs RC-High | 6 | 0.84 | 1 | -0.17 |
|  | RC-Medium vs Normal | 6 | 0.87 | 1 | -0.15 |
|  | RC-High vs Normal | 6 | 0.82 | 1 | -0.17 |
|  | RC-Medium vs RC-High | 6 | 0.81 | 1 | -0.19 |

**Table S7. The significant metabolic differentiators of RC treatment**

| **Name** | **mz** | **rt** | **ppm** | **formula** | **P.value** | **FDR** | | **VIP** |
| --- | --- | --- | --- | --- | --- | --- | --- | --- |
| L-Serine | 106.0495 | 50.6 | 3.4802993 | C3H7NO3 | 3.25E-08 | 2.86E-06 | 1.482107 | |
| 12-KETE | 319.2256 | 540.3 | 3.6839151 | C20H30O3 | 2.00E-07 | 7.89E-06 | 1.5150141 | |
| Phosphohydroxypyruvic acid | 184.9851 | 485.3 | 2.8326686 | C3H5O7P | 2.19E-07 | 8.36E-06 | 1.3414632 | |
| L-Threonine | 120.0652 | 50.8 | 2.298751 | C4H9NO3 | 9.73E-07 | 2.14E-05 | 1.5008005 | |
| 15-KETE | 318.2151 | 512.5 | 13.827125 | C20H30O3 | 5.95E-06 | 0.0001022 | 1.523927 | |
| 6-Keto-prostaglandin F1a | 371.2588 | 400.4 | 2.2188177 | C20H34O6 | 7.18E-06 | 8.16E-05 | 1.3773464 | |
| Betaine | 116.9287 | 634.2 | 12.292309 | C5H11NO2 | 5.55E-05 | 0.0004597 | 1.4544217 | |
| 11b-PGF2a | 335.2216 | 462.6 | 1.8196918 | C20H34O5 | 8.23E-05 | 0.0005996 | 1.4190803 | |
| L-Tryptophan | 205.0966 | 167.9 | 2.5460802 | C11H12N2O2 | 8.84E-05 | 0.0005101 | 1.2587131 | |
| L-Aspartic acid | 134.0444 | 50.8 | 2.8050407 | C4H7NO4 | 9.39E-05 | 0.0005357 | 1.0525419 | |
| 12(S)-HpETE | 319.2244 | 441.7 | 5.558748 | C20H32O4 | 0.0001686 | 0.000831 | 1.1891698 | |
| 12-Keto-leukotriene B4 | 317.2098 | 357.7 | 3.7044542 | C20H30O4 | 0.0002833 | 0.0012413 | 1.3360194 | |
| 20-HETE | 319.2273 | 592.4 | 1.6414636 | C20H32O3 | 0.0006041 | 0.0025979 | 1.4915585 | |
| 12-Keto-tetrahydro-leukotriene B4 | 336.3096 | 593 | 0.5600087 | C20H32O4 | 0.0007062 | 0.0024907 | 1.51301 | |
| Prostaglandin F2a | 337.2361 | 381.3 | 8.7001362 | C20H34O5 | 0.001183 | 0.0037437 | 1.1274105 | |
| Prostaglandin B2 | 317.2092 | 458.1 | 7.0426709 | C20H30O4 | 0.0061285 | 0.0144172 | 1.2898602 | |
| Glyceric acid | 105.0195 | 47.5 | 1.6758792 | C3H6O4 | 0.008991 | 0.0218482 | 1.3947532 | |
| Prostaglandin I2 | 335.2202 | 345.9 | 2.3575316 | C20H32O5 | 0.0092898 | 0.0202862 | 1.4457798 | |
| 5,6-DHET | 321.2404 | 552.7 | 6.6430001 | C20H34O4 | 0.023724 | 0.0440444 | 1.2885818 | |
| Prostaglandin H2 | 335.2202 | 357.6 | 0.425892255 | C20H32O5 | 0.001033664 | 0.005246339 |  | |
| Prostaglandin G2 | 351.2137 | 389.4 | 0.046304965 | C20H32O6 | 0.012817779 | 0.034378223 | 1.813542114 | |

**Table S8. Top 20 pentapeptide(5 Peptides) Identified After In Vitro Gastric Digestion of RC**

| **Peptide** | **-10lgP** | **Area [Sample]** | **Length** |
| --- | --- | --- | --- |
| P.GPAEF.T | 43.42 | 67500000.00 | 5 |
| P.PGAPG.P | 30.57 | 64800000.00 | 5 |
| D.AGQPG.E | 24.35 | 43900000.00 | 5 |
| D.KGETG.E | 36.16 | 27500000.00 | 5 |
| S.PGPAE.F | 30.3 | 26900000.00 | 5 |
| G.SPGPA.G | 28.72 | 22400000.00 | 5 |
| A.IGSPG.P | 36.9 | 22300000.00 | 5 |
| F.TAGNT.G | 39.68 | 20800000.00 | 5 |
| S.GPKGD.A | 35.19 | 14900000.00 | 5 |
| A.AGIKG.H | 28.7 | 13300000.00 | 5 |
| D.RGENG.S | 26.57 | 11900000.00 | 5 |
| G.NPGSD.G | 35.6 | 11600000.00 | 5 |
| S.PGAQG.P | 28.79 | 8330000.00 | 5 |
| G.SPGAQ.G | 33.82 | 7670000.00 | 5 |
| A.GITGA.R | 31.66 | 6390000.00 | 5 |
| G.EPGRD.G | 28.24 | 5770000.00 | 5 |
| G.SPGAQ(+.98).G | 30.01 | 4940000.00 | 5 |
| L.SGERG.P | 25.3 | 4520000.00 | 5 |
| A.GQQGA.I | 27.32 | 4480000.00 | 5 |
| G.VSGPK.G | 35.54 | 4140000.00 | 5 |

Note: -10lgP is the credibility of the peptide sequence identification from the corresponding mass spectrometry spectrum. Area [Sample] is the intensity values of the peptide segments in different samples are used for relative quantitative comparison.

**Table S9. Top 5 Peptides Identified After In Vitro Gastric Digestion of RC Protein**

| **Peptide** | **-10lgP** | **Area [Sample]** | **Length** |
| --- | --- | --- | --- |
| F.TAGNTGAPGSPGVSGPKGD.A | 74.85 | 7760000000.00 | 19 |
| E.FTAGNTGAPGSPGVSGPKGD.A | 72.94 | 2230000000.00 | 20 |
| A.IGSPGPAEF.T | 62.16 | 2070000000.00 | 9 |
| F.TAGNTGAPGSPGVSGPKG.D | 70.49 | 887000000.00 | 18 |
| F.TAGNTGAPGSPGVSGPKGDAGQPGEKG.S | 78.27 | 596000000.00 | 27 |

**Table S10. Sequences and design rationale of wild-type and G4-disrupting mutant *Pla2g4a* oligonucleotides**

| **Sequence name** | **Composition (5’-3’)** | **Length** |
| --- | --- | --- |
| *Pla2g4a*-G4-WT | GGGATGAGGGGCAGGGGGAGGG | 22 |
| *Pla2g4a*-G4-MutA | AAGATGAAAAGCAAAAGGAAAG | 22 |
| *Pla2g4a*-G4-MutT | TTGATGATTTGCATTTGGATTG | 22 |

**Table S11. Matrix-matched external calibration and quality assessment for GPAEF by targeted LC-MS/MS**

| **Nominal concentration (ng/mL)** | **Peak area** | **RT (min)** | **Back-calculated concentration (ng/mL)** | **Accuracy (%)** | **Calibration status** | **Note** |
| --- | --- | --- | --- | --- | --- | --- |
| 0.1 | 43 | 4.417 | 0.038 | 38.23 | Excluded | Instrument output was ND; one qualifier transition was not found. |
| 0.5 | 202 | 4.376 | 0.491 | 98.21 | Included | Lowest retained calibration level. |
| 1 | 303 | 4.295 | 0.779 | 77.87 | Excluded | Back-calculation acceptance criterion was not met. |
| 2 | 774 | 4.295 | 2.120 | 106.01 | Included | — |
| 5 | 1843 | 4.255 | 5.165 | 103.29 | Included | — |
| 10 | 3512 | 4.241 | 9.918 | 99.18 | Included | — |
| 20 | 7215 | 4.241 | 20.464 | 102.32 | Included | — |
| 50 | 17063 | 4.214 | 48.511 | 97.02 | Included | — |
| 100 | 33024 | 4.201 | 93.968 | 93.97 | Included | — |

**Notes:** Calibration standards were prepared in blank gastric mucosal lavage matrix and analyzed using external calibration. The final calibration model used 1/x^2^-weighted linear regression: y = 351.124x + 29.576 (weighted R^2^ = 0.9980). The 0.1 and 1 ng/mL levels were excluded from the final regression.

**Table S12. Targeted LC-MS/MS results for GPAEF in gastric mucosal lavage samples following intragastric administration of RC**

| **Time (min)** | **Sample** | **RT (min)** | **Response** | **Qualifier 1 agreement (%)** | **Qualifier 2 agreement (%)** | **Back-calculated concentration (ng/mL)** | **Final call** |
| --- | --- | --- | --- | --- | --- | --- | --- |
| 0 | 1 | 4.147 | 0 | — | — | ND | ND |
| 0 | 2 | 4.120 | 0 | — | — | ND | ND |
| 0 | 3 | 4.241 | 0 | — | — | ND | ND |
| 15 | 1 | 3.593 | 0 | — | — | ND | ND |
| 15 | 2 | 3.985 | 0 | — | — | ND | ND |
| 15 | 3 | 4.268 | 0 | — | — | ND | ND |
| 30 | 1 | 4.106 | 3089 | 98.1 | 98.8 | 8.713 | Detected and quantified |
| 30 | 2 | 4.106 | 3265 | 103.8 | 104.4 | 9.214 | Detected and quantified |
| 30 | 3 | 4.106 | 2999 | 99.8 | 97.4 | 8.457 | Detected and quantified |
| 120 | 1 | 4.106 | 20 | 162.8 | 94.4 | low-intensity | below calibration range |
| 120 | 2 | 4.106 | 17 | 97.7 | 100.3 | low-intensity | below calibration range |
| 120 | 3 | 4.106 | 22 | 93.8 | 53.7 | low-intensity | below calibration range |
| 240 | 1 | 4.268 | 0 | — | — | ND | ND |
| 240 | 2 | 4.093 | 0 | — | — | ND | ND |
| 240 | 3 | 4.255 | 0 | — | — | ND | ND |

**Notes:** Back-calculated concentrations were obtained from the final 1/x^2^-weighted calibration model shown in Table S11. GPAEF was consistently detected and quantified in three injections of the 30-min sample (mean ± SD = 8.795 ± 0.385 ng/mL; technical-injection CV = 4.38%). The responses observed at 120 min (17-22) were lower than the response of the lowest retained calibration standard (202) and did not consistently meet qualifier-ion criteria; the 120-min sample was therefore classified as low-level; not quantified.

**Figure S1 Infrared identification comparison between RC and porcine collagen**

**Figure S2. Effects of ethanol concentration gradients on GES-1 cell viability (1 h exposure)**

**Figure S3. Correlation between reactive oxygen species (ROS) levels and mitochondrial membrane potential (MMP) depolarization**

**Figure S4. RC-induced restoration of cellular morphology in ethanol-treated GES-1 cells (scale bar: 50μm)**

**Figure S5. Quantitative assessment of gross and histological gastric mucosal injury after ethanol-induced AGMI**

Quantitative evaluation of gastric mucosal injury in different treatment groups. (A) Number of bleeding points, (B) length of bleeding bands, and (C) width of bleeding bands. (D) Gross visual observation score and (E) the corresponding heat map summarizing the visual injury scores. (F) Histological observation score and (G) the corresponding heat map summarizing the histological injury scores. Data are presented as mean ± SD (n = 4 biological replicates per group). Statistical significance is indicated as #P < 0.05, ##P < 0.01, ###P < 0.001, and ####P < 0.0001 compared with the CK group; and *P < 0.05, **P < 0.01, ***P < 0.001, and ****P < 0.0001 compared with the Model group.

**Figure S6. Calibration and kinetic validation of biochemical assays used to assess gastric tissue redox status**

(A) Time-dependent absorbance profiles at 412 nm for total glutathione standards ranging from 0.5 to 15 μM. (B) Time-dependent absorbance profiles at 412 nm for GSSG standards ranging from 0.5 to 15 μM. (C) Final calibration curve relating GSSG concentration to the reaction rate (ΔOD412/min). (D) Final calibration curve relating total glutathione concentration to the reaction rate (ΔOD412/min). (E) MDA calibration curve based on absorbance at 532 nm. (F) Representative A340 kinetic traces used for GPx activity determination in the blank control and gastric tissue homogenates from the Normal, Model, RC High-dose, and commercial fish collagen groups.

**Figure S7. Calibration curves for the targeted quantification of PGE2 and 6-keto-PGF1α by competitive ELISA**

(A) Standard calibration curve for PGE2 quantification. (B) Standard calibration curve for 6-keto-PGF1α quantification. Absorbance was measured at 450 nm and plotted against the logarithm of the corresponding analyte concentration. Calibration relationships were established using logit-log regression, in which the logit-transformed response was fitted against the natural logarithm of analyte concentration. The fitted equations and coefficients of determination are shown in the corresponding panels. Data are presented as mean ± SD of technical replicates.

**Figure S8. Standard calibration curves for total cPLA2 and p-cPLA2 quantification by ELISA**

(A) Standard calibration curve for total cPLA2 quantification. Absorbance at 450 nm was plotted against cPLA2 concentration, and linear regression yielded the equation Y = 0.001606X − 0.05414 with R² = 0.9934. (B) Standard calibration curve for p-cPLA2 quantification. Absorbance at 450 nm was plotted against p-cPLA2 concentration, and linear regression yielded the equation Y = 0.00233X + 0.07730 with R² = 0.9925. Error bars represent the SD of technical replicates.

**Figure S9. The score plots of principal component analysis between the control, model** **or RC-Medium/High groups (n = 6 per group)**

**Figure S10. The differential metabolites heat map of RC-high vs Model vs Normal group**

**Figure S11. Changes in RNA expression levels of inflammatory and oxidative stress marker genes in gastric mucosal tissues**

**Figure S12. SDS-PAGE (left) and Western Blot (right) of RC after in vitro gastric digestion.**

Lane 1: Marker. Lane 2: Pepsin control. Lane 3: RC control. Lane 4: RC, digestion for 0 s. Lane 5: RC, digestion for 15 s. Lane 6: RC, digestion for 2 min. Lane 7: RC, digestion for 30 min. Lane 8: RC, digestion for 60 min.

**Figure S13. Intrinsic CD characterization and concentration optimization of representative RC-derived pentapeptides**

(A) Predicted three-dimensional structural model of GPAEF. (B) Concentration-dependent CD spectra of GPAEF at 0.1, 0.2, 0.5, and 1.0 mg/mL. (C) Corresponding high-tension voltage profiles recorded during CD acquisition. (D) Intrinsic CD spectra of the representative RC-derived pentapeptides GPAEF, KGETG, PGPAE, PGAPG, and AGQPG under identical experimental conditions. These measurements were used to evaluate intrinsic peptide spectral contributions and to select a peptide concentration compatible with a valid CD scanning range.

**Figure S14. CD characterization of wild-type and G4-disrupting mutant *Pla2g4a* sequences**

CD spectra of wild-type *Pla2g4a*-G4, G4-mutA, and G4-mutT under (A) Tris-KCl and (B) Tris-HCl conditions. Substitutions within the G-tracts markedly altered or weakened the characteristic CD features of the wild-type sequence, supporting impaired G4 folding by the mutant oligonucleotides.

**Figure S15. Conformational landscapes and corresponding clean energy profiles under distinct folding patterns**

**Figure S16. Analytical characterization and matrix-matched LC-MS/MS calibration of GPAEF.**

(A) Analytical HPLC chromatogram of the synthetic GPAEF standard, showing a major peak at 11.898 min and a purity of 98.416%. (B) Mass spectrum of synthetic GPAEF showing the expected protonated molecular ion at m/z 520.20 ([M+H]⁺). (C) Matrix-matched external calibration curve constructed using the retained calibration levels of 0.5, 2, 5, 10, 20, 50, and 100 ng/mL. The weighted linear regression equation was y = 351.1x + 29.58, with R² = 0.9980. The 0.1 and 1 ng/mL levels were not included in the final regression because they did not meet the identification or back-calculation criteria. (D) MRM chromatograms of the blank gastric lavage matrix, in which GPAEF was not detected. (E,F) MRM chromatograms of the 0.5 and 10 ng/mL GPAEF calibration standards, respectively. The transition m/z 520.2 → 155.0 was used for quantification, whereas m/z 520.2 → 355.0 and m/z 520.2 → 166.1 were used as qualifier transitions. ND, not detected.

**Figure S17. Targeted LC-MS/MS assessment of GPAEF in gastric lavage samples after oral RC administration.**
(A) MRM chromatograms and qualifier-ion profiles obtained from three injections of the 30-min gastric lavage sample. The quantifier transition (m/z 520.2 → 155.0) and the two qualifier transitions (m/z 520.2 → 355.0 and m/z 520.2 → 166.1) coeluted at 4.106 min and exhibited consistent ion-ratio agreement, supporting the identification of GPAEF. (B) Three injections of the 120-min sample showed low-intensity GPAEF-related signals near the expected retention time; however, these signals did not consistently satisfy the predefined qualifier-ion and quantitative criteria and were therefore not reported as confirmed or quantified GPAEF. (C-E) Representative MRM chromatograms of the 0-, 15-, and 240-min samples, respectively, in which GPAEF was not detected under the present analytical conditions. Black, quantifier transition m/z 520.2 → 155.0; blue and green, qualifier transitions m/z 520.2 → 355.0 and m/z 520.2 → 166.1, respectively. ND, not detected under the present analytical conditions.

**Figure S18. MD simulation of the mixed G4‑GPAEF complex demonstrates its intrinsic stability.**

(A) Time evolution of root‑mean‑square deviation (RMSD) for the mixed G4‑GPAEF complex. (B) Residue‑level root‑mean‑square fluctuation (RMSF) of the complex. (C) Temporal change in the radius of gyration (Rg). (D) Evolution of the intermolecular hydrogen‑bond network between GPAEF and G4. (E) MM-PBSA binding free energy decomposition for the mixed G4‑GPAEF complex.

**Figure S19. G4-induced DNA pre-melting facilitates transcription initiation**

**Figure S20. Molecular Dynamics Analysis of the Mixed-Topology *Pla2g4a*-G4/GPAEF Complex.**

**Figure S21. Temperature-dependent liquid-gel phase transition of RC**
